# Supplementary material for: Childhood peer status and circulatory disease in adulthood: a prospective cohort study in Stockholm, Sweden
Source: BMJ Open. 2020 Sep 15;10(9):e036095. doi: 10.1136/bmjopen-2019-036095 (PMC7493119; doi:10.1136/bmjopen-2019-036095)
Supplement: Author's manuscript [file bmjopen-2019-036095.draft_revisions.pdf]

BMJ Open is committed to open peer review. As part of this commitment we make the peer review history of every article we publish publicly available.

When an article is published we post the peer reviewers' comments and the authors' responses online. We also post the versions of the paper that were used during peer review. These are the versions that the peer review comments apply to.

The versions of the paper that follow are the versions that were submitted during the peer review process. They are not the versions of record or the final published versions. They should not be cited or distributed as the published version of this manuscript.

BMJ Open is an open access journal and the full, final, typeset and author-corrected version of record of the manuscript is available on our site with no access controls, subscription charges or pay-per-view fees (http://bmjopen.bmj.com).

If you have any questions on BMJ Open's open peer review process please email info.bmjopen@bmj.com

BMJ Open

Childhood peer status and risk for circulatory disease in adulthood: a prospective cohort study

Journal:	*BMJ Open*
Manuscript ID	bmjopen-2019-036095
Article Type:	Original research
Date Submitted by the Author:	02-Dec-2019
Complete List of Authors:	Miething, Alexander; Stockholms Universitet, Department of Public Health Sciences<br>Almquist, Ylva; Stockholm University, Department of Public Health Sciences
Keywords:	EPIDEMIOLOGY, PUBLIC HEALTH, STROKE MEDICINE

SCHOLARONE™
Manuscripts

Title:

Childhood peer status and risk for circulatory disease in adulthood: a prospective cohort study

Authors:

Alexander Miething and Ylva Brännström Almquist

Affiliations

Department of Public Health Sciences, Stockholm University, SE-106 91, Stockholm, Sweden.

Corresponding author:

Alexander Miething

E-mail: alexander.miething@su.se

For peer review only

For peer review only - http://bmjopen.bmj.com/site/about/guidelines.xhtml

Abstract

Objectives: Childhood conditions have been recognised as important predictors of short-term and long-term health outcomes, but few studies considered status position in the peer group as a possible determinant of adult health. Lower peer status, which often reflects experiences of marginalization and rejection by peers, may impose inequality experiences and leave long-lasting imprints on health. The present study therefore aimed to examine whether peer status contributes to the risk for circulatory disease across adulthood.

Design: Prospective cohort study based on the Stockholm Birth Cohort Multigenerational Study (SBC Multigen).

Setting: Stockholm metropolitan area

Participants: All individuals who were born in 1953 and resident in the greater metropolitan area of Stockholm in 1963 (n=15,117). The analytical sample consisted of 5410 males and 5990 females. Peer status was sociometrically assessed when cohort members were 13 years old. The survey material was linked to inpatient-care registers that contained information about circulatory diseases across ages 20-63. Cox proportional hazard models were used for the analysis.

Outcome measures: Circulatory disease

Results: Peer marginalisation at age 13 showed significantly higher risks of circulatory disease in adulthood in males (HR=1.34; 95% CI 1.10–1.64) and females (HR=1.36; 95% CI 1.07–1.74). A graded relationship between peer status and circulatory diseases was detected in females (p=0.012). Among males, there was a threshold effect, showing that only those in the lowest status position had significantly increased risks of circulatory disease. The associations remained significant after adjusting for various conditions in child- and adulthood.

Conclusions: This study demonstrated that circulatory diseases in adulthood may be traceable back to low peer status and marginalisation in childhood. It is suggested that peer status in late childhood may precede social integration in adolescence and adulthood, acting as long-term stressors contributing to circulatory disease through biological, behavioural, and psychosocial pathways.

For peer review only

For peer review only - http://bmjopen.bmj.com/site/about/guidelines.xhtml

Strengths and limitations of this study

- The longitudinal data material with linkages to register data allowed us to study the long-term implications of childhood conditions for health outcomes in adulthood.
- High response rates minimised the effects of selection and biases from non-response.
- Peer status in childhood as well as other covariates were assessed at a single time point.
- There was little information available about health and health behaviours in childhood and across the subsequent life course that might confound the demonstrated associations.

Keywords

Peer status; Childhood; Circulatory disease; In-patient care; Life course; Birth cohort

For peer review only

For peer review only - http://bmjopen.bmj.com/site/about/guidelines.xhtml

Declarations

Acknowledgement

This work was supported by the Swedish Council for Health, Working Life and Social Research [grant no. 2016-07148], (http://www.forte.se/en/).

Authors' contributions

AM and YBA conceived this study. YBA prepared and compiled the data. AM performed the statistical analyses. AM and YBA wrote the manuscript. Both authors approved the final version of the manuscript.

Competing interests

The authors declare that they have no competing interests.

Availability of data and materials

The datasets generated and/or analysed during the current study are not publicly available due to ethical regulations regarding the Stockholm Birth Cohort (SBC) study but are available from the co-author on reasonable request.

Ethics approval and consent to participate

The study was approved by the Stockholm Regional Ethics Committee (reg. no. 2016/481-31/5; 2016/481-31/5). The need for consent was waived due to the retrospective nature of this study.

For peer review only

Introduction

Innumerable studies have shown the importance of childhood conditions for later life outcomes and, within health inequality research, parental socioeconomic conditions is often put forward as a key determinant for health in adulthood (Cohen et al., 2010; Galobardes, 2004; Poulton et al., 2002). For example, adverse socioeconomic circumstances in childhood have been shown to predict poor mental health, but also metabolic problems, cardiovascular diseases, and higher risks of mortality (Danese et al., 2009; Galobardes et al., 2006; Smith et al., 1998). Children nevertheless also hold positions in status structures outside the family, such as in their peer group and school contexts. Yet, relatively few studies have addressed how experiences related to these childhood-specific types of social structure are related to health outcomes in later life (Almquist, 2009; Caspi et al., 2006). The present study therefore aimed to investigate the long-term implications of the child's own social position – peer status – for the risk of circulatory disease, which represents one of the leading causes for mortality.

Peer orientation significantly increases in early adolescence (Fuligni et al., 2001). Seeking for greater autonomy, children distance themselves from their parents and simultaneously intensify their interactions with peers (Fuligni and Eccles, 1993; Starfield et al., 2002). This entails that the family context become less important, whereas the role of peer- and school-related factors become increasingly important. Intensified peer orientation has also been hypothesised to have buffering effects on socioeconomic inequalities in health. In conjuncture with the "processes of equalisation" hypothesis, the socioeconomic gradient in health tends to attenuate during the child–youth transition, while experiences related to peer interactions and youth culture become more salient (West and Sweeting, 2004). A plausible explanation is that peer interactions and school environment impose a homogenising effect on children's background characteristics and supersede the repercussions of children's socioeconomic origin. Instead of socioeconomic conditions, the peer context and children's position in the peer group emerge as important determinants of health and the adoption of health behaviours. Several studies have shown that the initiation of health-related behaviours, such as smoking, is substantially driven by peers rather than determined by socioeconomic conditions (Hanson and Chen, 2007; Kobus, 2003; Richter and Lampert, 2008).

Peers function as socialising agents and are important for children's social, emotional, and behavioural development. Interactions with peers facilitate experiences that parents cannot supply (Bagwell et al., 1998), as well as contribute to identity formation (Brown et al., 1986) and personality maturation (Reitz et al., 2014). Furthermore, these interactions have the potential to increase children's self-worth, the ability to build relationships in later life, and adaptive adjustment (LaFontana and Cillessen,

For peer review only - http://bmjopen.bmj.com/site/about/guidelines.xhtml

2002; Parker and Asher, 1987). Children are usually well aware of the advantages related to higher status in the peer group (Lease et al., 2002). Moreover, their concerns about popularity and reputation significantly increase when they approach adolescence (Fournier, 2009). These affection-based dimensions play an important role in the formation of sociometric peer status, which represents an established instrument to assess the degree to which child is a liked, accepted, and integrated member of the peer group (Almquist, 2009; Parkhurst and Hopmeyer, 1998). Marginalisation and peer rejection – conditions that are reflected by a low peer status position – deprive children of experiences that are important for their subsequent emotional and cognitive development, and may thus increase the risk for poor well-being and reduced mental health (Oldehinkel et al., 2007). Östberg and Modin (2008) demonstrated a graded inverse relationship between peer status and long-standing illness. Accordingly, although disadvantages arise most clearly for children in the bottom group, they are also tangible for those with higher ranks in the peer hierarchy. However, it is rather obvious that marginalised and socially isolated children suffer most as they are exceedingly vulnerable to bullying and other forms destructive peer interactions (Wolke et al., 2013). Because (pre)adolescence coincides with an increased developmental need and strong desire for peer affiliations, peer rejection and marginalisation may not only have pervasive impacts on current well-being and health, but also affect children's ability to engage in social relationship later on (Lease et al., 2002; Östberg and Modin, 2008; Plenty and Jonsson, 2017). As acknowledged through the theory of cumulative disadvantages (Dannefer, 2003; DiPrete and Eirich, 2006), adverse conditions and negative experiences in childhood tend to accrue across the life course, and magnify detriments in later health as well (Shuey and Willson, 2014).

Among adults, greater social integration has been linked to reduced risks of hypertension, cardiovascular diseases, obesity, and mortality (Cundiff and Matthews, 2018; House et al., 1988; Valtorta et al., 2016), indicating that psychosocial mechanisms are at play. It is reasonable to assume that similar processes link children's peer status to later circulatory diseases (Kroenke, 2008). Higher status positions in the peer group enable children to derive greater emotional, cognitive, and social support from social contacts and facilitate the access to tangible and non-tangible resources provided by social contacts. In line with the buffering hypothesis of social support (Cohen and Wills, 1985), supportive social ties impose indirect protective effects on health as they enhance individuals' coping ability and moderate their neuroendocrine or behavioural responses to acute or chronic stress (Holt-Lunstad et al., 2010; Seeman, 1996; Umberson and Karas Montez, 2010). High peer status may also directly enhance individuals' self-care, achievements, and health behaviours – factors that mediate the risk to develop circulatory diseases in adulthood (Uchino, 2006). Confounding factors may nevertheless induce spurious associations between peer status and circulatory disease in later life.

For peer review only - http://bmjopen.bmj.com/site/about/guidelines.xhtml

Plausible confounding variables are aggressive or controversial behaviours and lower cognitive abilities that are associated with peer rejection as well as poor adult health. Likewise, family-related circumstances such as number of siblings and birth order/sibling position possibly influence children's standing in the peer group, and may also impose long-term effects on health.

Based on data from a Swedish cohort born in 1953, who have been followed for more than 60 years, the present study examines the association between sociometrically assed peer status in childhood (age 13) and circulatory disease during adulthood (ages 20-63). Drawing on earlier studies which have shown an association between social status inequalities and increased risks for cardiovascular and circulatory diseases through psychosocial mechanisms in the adult population, an inverse association between peer status and circulatory diseases is hypothesised. The register-linked survey material with its extensive follow-up, provides a rich set of variables to study the role of potential confounding and mediating factors.

Data and methods

The data material used in the current study was the Stockholm Birth Cohort Multigenerational Study (SBC Multigen). In 2018/2019, the SBC Multigen was established through a probability matching between two longitudinal, anonymised data materials. The first was the Stockholm Metropolitan Study (SMS), defined as all individuals who were born in 1953 and resident in the greater metropolitan area of Stockholm in 1963 (n=15,117). It encompasses survey and register information collected continuously from 1953 up until 1986, after which data were anonymised. The second was RELINK53, defined as all individuals born in 1953 who lived in Sweden in 1960, 1965, and/or 1968, as well as their ascendant, contemporaneous, and descendant family members (n=2,390,753). RELINK53 contains detailed information collected from a variety of administrative registers between 1960 and 2018. An algorithm based on a set of variables present in both data materials, rendered it possible to match 14,608 of the SMS cohort members to RELINK53 (for more information, see Almquist et al., Forthcoming). Accordingly, we are able to follow these individuals from birth up until their 60s. The Regional Ethical Review Board in Stockholm approved the creation of RELINK53 as well as the probability matching to the SMS that resulted in SBC Multigen (no. 2017/34-31/5; 2017/684-32).

Patient and Public Involvement

The data in this study were retrospectively compiled. Therefore, patients or the public were to no time involved in the design of this study.

Dependent variable

For peer review only - http://bmjopen.bmj.com/site/about/guidelines.xhtml

Information on circulatory diseases was drawn from the Swedish hospital discharge register, covering the years 1973 to 2016. This register records all in-patient care events (with overnight stays) in Swedish hospitals. In this study, circulatory diseases comprised all diagnoses included in the International Classification of Diseases (ICD 10th revision; chapter IX (I00 – I99)). The diagnoses were primarily based on judgments of medical doctors. Preceding versions of the ICD (8th and 9th revision) apply to diagnoses made from 1973 to 1986, and 1987 to 1996 respectively. These older classifications were harmonised in accordance with the 10th revision of the ICD.

Explanatory variable

Peer status was measured through the question "Whom do you best like working with at school?" (Almquist, 2009, 2011). Students were asked to nominate three classmates, in no particular order. This sociometric assessment was conducted with all 6-grade students in the Stockholm metropolitan area in 1966. An exception were students with learning disabilities. For the purpose of the current study, we additionally excluded those who attended school classes with less than 10 children, since the peer status distribution tends to be somewhat different in small classes. Based on the number of received nominations, four categories were created: "marginalised" denoted zero nominations, "low status" one nomination, "medium status" two or three nominations, and "high status" four or more nominations.

Covariates

The covariates considered family-related, school-related, and individual circumstances and conditions. The family-related factors considered the presence of siblings, parents' education and socioeconomic conditions, parents' psychiatric problems, and conditions during delivery. Number of siblings and sibling position were assessed in 1972. The latter measured whether the study participant was the only child in the family, or whether he/she was last born, middle born, or first born. Parental educational level accounted for whether there was at least one adult in the household with an upper secondary school degree. Parents' receipt of social welfare benefits accounts for socioeconomic difficulties and was available from the social registers, covering the period 1953 to 1965. Parental psychiatric problems (available from 1953 to 1972) were derived from the social registers and measured whether fathers or mothers showed symptoms of mental illness or psychiatric problems. Information on intervention during the delivery was based on delivery records. Cognitive ability can be regarded as individual-level confounder that affects a child's position in the peer groups but also relates to (predicts) adverse health behaviours and the risk of poor health in later life. A test of cognitive ability was conducted in 1966 and measured participants' verbal, spatial, and numerical competences. The test scores were grouped into quartiles of cognitive ability. Average school marks

For peer review only - http://bmjopen.bmj.com/site/about/guidelines.xhtml

measured study participants' school performance in the 6th grade. Average conduct marks assessed in the 6th grade and crimes of violence recorded between 1962 and 1972 indicate participants' externalising problems. Similar to school marks, conduct marks and violent behaviour may both mediate and confound the hypothesised association between peer status and circulatory disease.

Study population

The study population included all cohort members in the SBC Multigen who attended school classes with at least 10 children, and had complete information on the variables of interest. Accordingly, the analytical sample resulted in 5,410 males and 5,990 females.

Statistical methods

Cox proportional hazard models were used to estimate the risk of circulatory disease. As information on circulatory morbidity was available from 1973 to 2016, study subjects were left-truncated at 1 January 1973. Right-censoring was applied when the observation ended on 31 December 2016, or earlier, if subjects had died from any disease.

Results

The descriptive statistics are shown in Table 1 and illustrate a higher prevalence of circulatory diseases for males (18.5 %) as compared to females (11.2 %). The distribution of peer status is relatively similar across the genders, although a slightly higher proportion of males (31.1 %) than females (28.6 %) were in high status positions, at the same time as more females (15.7 %) than males (12.1 %) experienced marginalisation.

< Table 1 about here >

The results from the Cox proportional hazard models are shown in Table 2 for males and Table 3 for females. The unadjusted hazard ratios of males' peer status in Model 1 (Table 2) display that only those who are in a marginalised position have an increased risk for circulatory disease (HR=1.54; 95% CI 1.27–1.87). Controlling for sibling status and number of siblings in Model 2 does not affect the hazards of peer status. When adding parental educational level and social welfare receipt to the model, a small decrease of the hazard ratio of marginalised peer position can be observed (Model 3). A likelihood ratio test confirmed that the decrease is significant compared to Model 2. The estimates are further attenuated when we control for parental psychiatric problems, intervention during delivery, and cognitive ability in Model 4. The fully controlled Model 5 additionally considers conduct

For peer review only - http://bmjopen.bmj.com/site/about/guidelines.xhtml

marks, average school marks, and crimes of violence, but still retains a significant association between marginalisation and circulatory disease.

< Table 2 about here >

Table 3 demonstrates the corresponding analysis for females. Model 1 illustrates a graded association between peer status and circulatory disease. Females with medium peer status have a 28% higher risk of experiencing a circulatory disease as compared to high status females. The respective increase by low peer status is 42%, and 61% for females in a marginalised position, compared to females in a high status position. As shown for males, a gradual attenuation of the hazard ratios of peer status can be observed when control variables are successively added to the model. In Model 4, the hazard ratio for medium status is no longer significant, whereas in Model 5, only marginalised females show a significant risk for circulatory disease (HR=1.36; 1.07–1.74). Despite the non-significant estimates of medium and low status, a significant trend in the negative association between peer status and circulatory disease by can still be detected (p=0.012).

< Table 3 about here >

Whereas females' medium and low status positions indicate larger relative risks of circulatory diseases compared to males, marginalised peer status reveals remarkably similar estimates for males and females. It is further noteworthy that the considered covariates demonstrate different associations with circulatory disease in males and females. Family circumstances (including sibling position, number of siblings, parental educational level, and parental psychiatric problems) show significant associations with circulatory disease in females only. Exceptions are parental social welfare receipt and interventions during delivery, which demonstrate significant associations with the outcome in males only. Low cognitive ability and low conduct marks predict circulatory disease in in males but not in females. Low school marks seem to matter more for females. Crimes of violence poses a risk factor in both males and females, though with a notably higher magnitude in females.

Discussion

The present study demonstrated that peer status in preadolescence is associated with circulatory diseases in adulthood. More specifically, being in a marginalised position in the peer group is significantly associated with increased risks of circulatory disease in both males and females. In addition, a graded association is detected for females, suggesting that their risks of circulatory disease decrease by a gradual improvement of position in the peer group. Compared to the most popular females, even those with a medium-high position in the peer group run higher risks of circulatory

For peer review only - http://bmjopen.bmj.com/site/about/guidelines.xhtml

diseases in later life. The corresponding analyses of males revealed a threshold effect. Only socially isolated (marginalised) males displayed significant hazards of circulatory disease. The demonstrated associations remained significant also after controlling for variables that could be regarded as potential confounders, including family-related factors and socioeconomic circumstances. Neither did the variables here considered as mediators (school marks, conduct marks, and crimes of violence) reduce the strength of association to any substantial extent. The absence of mediating and confounding factors could suggest an independent association between peer status and circulatory disease, which is in line with other studies that considered long-term impacts of peer integration (Almquist, 2009; Cundiff and Matthews, 2018; Danese et al., 2009; Gustafsson et al., 2012). However, since this remains to be confirmed with data materials that also encompasses additional types of confounding and mediating factors.

Past research has argued that peer status may be considered as a childhood-specific type of social structure that in many ways resemble social status positions in adult life (Almquist, 2009, 2011). Accordingly, it is plausible that lower peer status would have health implications similar to socioeconomic deprivation and low socioeconomic position in adulthood. As also outlined earlier in this study, peer status may even equalise the effects of low parental socioeconomic position (West and Sweeting, 2004). Not least, the fact that the estimates for peer status in this study are equivalent in strength compared to childhood socioeconomic disadvantages, supports this interpretation.

The current findings contribute to a growing research field focussing on the long-lasting impacts of childhood circumstances on health (Caspi et al., 2006). Studies on health implications of adults' social relationships, however, tend to disregard the emergence of socially induced health problems and the origin of destructive social experiences at earlier stages in life (Ehrlich 2015). In line with life course theory, deficient social relationships – as well as health problems – may be the result of cumulative processes that escalate with increasing age. Accordingly, earlier research conjectured a mutual interdependence of social relationships and health at any stages in life course (Östberg and Modin, 2008). The results found in the current study suggest that this process can be backtracked to adolescence and childhood.

Among adults, stress-buffering effects of tangible and non-tangible support have been proposed as primary mechanisms as to why constructive social relationships are beneficial for health (Cohen and Wills, 1985; Uchino, 2006). Low peer status, social isolation, and marginalisation may be particularly distressing in childhood. Socially isolated children suffer not only from lacking social and emotional support but also from the experience of having reduced opportunities to make friends, to control dominating behaviour by others, and run great risk to be victimised and bullied (Oldehinkel et al.,

For peer review only - http://bmjopen.bmj.com/site/about/guidelines.xhtml

2007). The limited coping capabilities of children may reinforce these negative consequences and increase distress. In addition, these adversities might disturb children's social and emotional development and lead to behaviours and disadvantages that are difficult to rectify in their later life.

Low peer status in childhood and adolescence has been identified as source of chronic (psychosocial) stress that furthers the development of mental health problems and chronic inflammation (Danese et al., 2009; Nurius et al., 2015; Yang et al., 2013). In adulthood, lacking social contacts are associated with disorders that develop over a long time span, including metabolic problems, hypertension, cardiovascular problems, and stroke. Earlier research on childhood conditions and life course processes (Hayward and Gorman, 2004; Miller et al., 2011; Power and Hertzman, 1997), as well as the findings of the current study, suggest that the childhood perspective on the one hand, and adulthood perspective on the other hand, should not be considered as independent given that socially induced morbidity and mortality in adulthood may have their origin in childhood. There is convincing evidence from neuroscience regarding how social relationships modulate neuroendocrine responses that subsequently affect the circulatory system, increasing the risk for stroke and cardiovascular diseases (Booth et al., 2015). Similar mechanisms may apply to the findings in the current study and explain why marginalisation in the peer group poses a risk factor for the development of circulatory diseases.

Another finding worth noting are the observed differences in associations between males and females. Similar to our findings, previous research have detected a dose-response relationship between females' peer status and metabolic syndrome in adulthood (Gustafsson et al., 2012). The graded associations between peer status and circulatory disease in females could suggest a greater emotional and social reactivity to chronic stress in comparison to males (Rose and Rudolph, 2006; Rudolph, 2002). Girls' greater vulnerability to interpersonal stress may further be reinforced by gender differences in social interactions and acquisition of social status in the peer group. Whereas girls tend to be relationship-oriented, boys are more involved in competitive and organised play (Rose and Rudolph, 2006). Boys are also more concerned about achievements and problems directly affecting themselves (Shih et al., 2006). These differences become manifest in girls' greater worries and self-blaming about others' relationship problems and concerns about negative evaluation by other peers. Because girls pay more attention to their peer environment and social interactions (Sneed & Cohen 2014), they may react more strongly to feedback from peers. In contrast, boys' stress pertains to school performance, physical competition, and close friendships rather than to their social networks. According to Oldehinkel (2007), likability and affection can be considered as the primary criteria of high peer status in girls, while admiration (of their achievements) marks the status position of boys. With regard to our findings, it can be concluded that not belonging to the most liked (i.e., the most popular peers) may hurt more than not belonging to the most admired.

For peer review only - http://bmjopen.bmj.com/site/about/guidelines.xhtml

Limitations

A particular strength of this study is the use of longitudinal data material with linkages to register data that allowed us to study the long-term implications of childhood conditions for health outcomes in adulthood. Response rates were relatively high, which minimised the effects of selection and biases from non-response. The studied single cohort covered a fairly homogeneous population. Therefore, certain spurious effects, such as influences on the presented associations due to immigration, can be ruled out. Nevertheless, this study is also subject to limitations. Peer status in childhood as well as other covariates were assessed at a single time point. Further, there was little information available about health and health behaviours in childhood and across the subsequent life course (i.e., in adolescence and adulthood) that might confound the demonstrated associations. Moreover, it was not possible to determine when health problems (e.g., the predecessors of circulatory diseases) emerged. Likewise, potentially relevant information about circumstances in adulthood (e.g. achievements and ambitions) was not available in the data. It was therefore not possible to fully identify the causal pathways, for instance, whether psychological, behavioural, or biological processes contributed to demonstrated associations between peer status and circulatory disease. Despite the use of controls for parental socioeconomic status and other confounders such as family circumstances, it is nevertheless possible that selection and unmeasured confounding affected the identified associations. Given these limitations, we cannot draw any causal inference from this study.

Conclusions

This study confirmed that childhood circumstances impose health effects in later life. Peer relations do not only play an important role for children's emotional and social development but may have considerable long-term implications on their health. Congruent with earlier studies on the role of socioeconomic conditions for morbidity and mortality, the findings of this study stress the importance of children's social relationships for circulatory diseases. Our investigating of this rather specific outcome might help to understand the processes that contribute to socially induced morbidity and mortality.

For peer review only - http://bmjopen.bmj.com/site/about/guidelines.xhtml

References

Almquist, Y., 2011. Social isolation in the classroom and adult health: A longitudinal study of a 1953 cohort. Adv. Life Course Res. 16, 1–12. https://doi.org/10.1016/j.alcr.2010.11.001

Almquist, Y., 2009. Peer status in school and adult disease risk: a 30-year follow-up study of disease-specific morbidity in a Stockholm cohort. J. Epidemiol. Community Health 63, 1028–1034. https://doi.org/10.1136/jech.2009.088377

Almquist, Y.B., Grotta, A., Vågerö, D., Stenberg, S.-Å., Modin, B., Forthcoming. Cohort Profile Update: The Stockholm Birth Cohort Study. Int J Epidemiol.

Bagwell, C.L., Newcomb, A.F., Bukowski, W.M., 1998. Preadolescent Friendship and Peer Rejection as Predictors of Adult Adjustment. Child Dev. 69, 140–153. https://doi.org/10.1111/j.1467-8624.1998.tb06139.x

Booth, J., Connelly, L., Lawrence, M., Chalmers, C., Joice, S., Becker, C., Dougall, N., 2015. Evidence of perceived psychosocial stress as a risk factor for stroke in adults: a meta-analysis. BMC Neurol. 15. https://doi.org/10.1186/s12883-015-0456-4

Brown, B.B., Eicher, S.A., Petrie, S., 1986. The importance of peer group (“crowd”) affiliation in adolescence. J. Adolesc. 9, 73–96. https://doi.org/10.1016/S0140-1971(86)80029-X

Caspi, A., Harrington, H., Moffitt, T.E., Milne, B.J., Poulton, R., 2006. Socially Isolated Children 20 Years Later: Risk of Cardiovascular Disease. Arch. Pediatr. Adolesc. Med. 160, 805. https://doi.org/10.1001/archpedi.160.8.805

Cohen, S., Janicki-Deverts, D., Chen, E., Matthews, K.A., 2010. Childhood socioeconomic status and adult health: Childhood socioeconomic status and adult health. Ann. N. Y. Acad. Sci. 1186, 37–55. https://doi.org/10.1111/j.1749-6632.2009.05334.x

Cohen, S., Wills, T.A., 1985. Stress, social support, and the buffering hypothesis. Psychol. Bull. 98, 310–357. https://doi.org/10.1037/0033-2909.98.2.310

Cundiff, J.M., Matthews, K.A., 2018. Friends With Health Benefits: The Long-Term Benefits of Early Peer Social Integration for Blood Pressure and Obesity in Midlife. Psychol. Sci. 29, 814–823. https://doi.org/10.1177/0956797617746510

Danese, A., Moffitt, T.E., Harrington, H., Milne, B.J., Polanczyk, G., Pariante, C.M., Poulton, R., Caspi, A., 2009. Adverse Childhood Experiences and Adult Risk Factors for Age-Related Disease: Depression, Inflammation, and Clustering of Metabolic Risk Markers. Arch. Pediatr. Adolesc. Med. 163. https://doi.org/10.1001/archpediatrics.2009.214

Dannefer, D., 2003. Cumulative Advantage/Disadvantage and the Life Course: Cross-Fertilizing Age and Social Science Theory. J. Gerontol. B. Psychol. Sci. Soc. Sci. 58, S327–S337. https://doi.org/10.1093/geronb/58.6.S327

DiPrete, T.A., Eirich, G.M., 2006. Cumulative Advantage as a Mechanism for Inequality: A Review of Theoretical and Empirical Developments. Annu. Rev. Sociol. 32, 271–297. https://doi.org/10.1146/annurev.soc.32.061604.123127

Fournier, M.A., 2009. Adolescent Hierarchy Formation and the Social Competition Theory of Depression. J. Soc. Clin. Psychol. 28, 1144–1172. https://doi.org/10.1521/jscp.2009.28.9.1144

Fuligni, A.J., Eccles, J.S., 1993. Perceived parent-child relationships and early adolescents’ orientation toward peers. Dev. Psychol. 29, 622–632. https://doi.org/10.1037/0012-1649.29.4.622

Fuligni, A.J., Eccles, J.S., Barber, B.L., Clements, P., 2001. Early adolescent peer orientation and adjustment during high school. Dev. Psychol. 37, 28–36. https://doi.org/10.1037//0012-1649.37.1.28

Galobardes, B., 2004. Childhood Socioeconomic Circumstances and Cause-specific Mortality in Adulthood: Systematic Review and Interpretation. Epidemiol. Rev. 26, 7–21. https://doi.org/10.1093/epirev/mxh008

For peer review only - http://bmjopen.bmj.com/site/about/guidelines.xhtml

Galobardes, B., Smith, G.D., Lynch, J.W., 2006. Systematic Review of the Influence of Childhood Socioeconomic Circumstances on Risk for Cardiovascular Disease in Adulthood. Ann. Epidemiol. 16, 91–104. https://doi.org/10.1016/j.annepidem.2005.06.053

Gustafsson, P.E., Janlert, U., Theorell, T., Westerlund, H., Hammarström, A., 2012. Do Peer Relations in Adolescence Influence Health in Adulthood? Peer Problems in the School Setting and the Metabolic Syndrome in Middle-Age. PLoS ONE 7, e39385. https://doi.org/10.1371/journal.pone.0039385

Hanson, M.D., Chen, E., 2007. Socioeconomic Status and Health Behaviors in Adolescence: A Review of the Literature. J. Behav. Med. 30, 263–285. https://doi.org/10.1007/s10865-007-9098-3

Hayward, M.D., Gorman, B.K., 2004. The Long Arm of Childhood: The Influence of Early-Life Social Conditions on Men's Mortality. Demography 41, 87–107. https://doi.org/10.1353/dem.2004.0005

Holt-Lunstad, J., Smith, T.B., Layton, J.B., 2010. Social Relationships and Mortality Risk: A Meta-analytic Review. PLoS Med. 7, e1000316. https://doi.org/10.1371/journal.pmed.1000316

House, J.S., Landis, K.R., Umberson, D., 1988. Social Relationships and Health. Science 241, 540.

Kobus, K., 2003. Peers and adolescent smoking. Addiction 98, 37–55. https://doi.org/10.1046/j.1360-0443.98.s1.4.x

Kroenke, C., 2008. Socioeconomic status and health: Youth development and neomaterialist and psychosocial mechanisms. Soc. Sci. Med. 66, 31–42. https://doi.org/10.1016/j.socscimed.2007.07.018

LaFontana, K.M., Cillessen, A.H.N., 2002. Children's perceptions of popular and unpopular peers: A multimethod assessment. Dev. Psychol. 38, 635–647. https://doi.org/10.1037//0012-1649.38.5.635

Lease, A.M., Musgrove, K.T., Axelrod, J.L., 2002. Dimensions of Social Status in Preadolescent Peer Groups: Likability, Perceived Popularity, and Social Dominance. Soc. Dev. 11, 508–533. https://doi.org/10.1111/1467-9507.00213

Miller, G.E., Chen, E., Parker, K.J., 2011. Psychological stress in childhood and susceptibility to the chronic diseases of aging: Moving toward a model of behavioral and biological mechanisms. Psychol. Bull. 137, 959–997. https://doi.org/10.1037/a0024768

Nurius, P.S., Green, S., Logan-Greene, P., Borja, S., 2015. Life course pathways of adverse childhood experiences toward adult psychological well-being: A stress process analysis. Child Abuse Negl. 45, 143–153. https://doi.org/10.1016/j.chiabu.2015.03.008

Oldehinkel, A.J., Rosmalen, J.G.M., Veenstra, R., Dijkstra, J.K., Ormel, J., 2007. Being Admired or Being Liked: Classroom Social Status and Depressive Problems in Early Adolescent Girls and Boys. J. Abnorm. Child Psychol. 35, 417–427. https://doi.org/10.1007/s10802-007-9100-0

Östberg, V., Modin, B., 2008. Status relations in school and their relevance for health in a life course perspective: Findings from the Aberdeen children of the 1950's cohort study. Soc. Sci. Med. 66, 835–848. https://doi.org/10.1016/j.socscimed.2007.10.018

Parker, J.G., Asher, S.R., 1987. Peer relations and later personal adjustment: Are low-accepted children at risk? Psychol. Bull. 102, 357–389. https://doi.org/10.1037/0033-2909.102.3.357

Parkhurst, J.T., Hopmeyer, A., 1998. Sociometric Popularity and Peer-Perceived Popularity: Two Distinct Dimensions of Peer Status. J. Early Adolesc. 18, 125–144. https://doi.org/10.1177/0272431698018002001

Plenty, S., Jonsson, J.O., 2017. Social Exclusion among Peers: The Role of Immigrant Status and Classroom Immigrant Density. J. Youth Adolesc. 46, 1275–1288. https://doi.org/10.1007/s10964-016-0564-5

Poulton, R., Caspi, A., Milne, B.J., Thomson, W.M., Taylor, A., Sears, M.R., Moffitt, T.E., 2002. Association between children's experience of socioeconomic disadvantage and adult health: a life-course study. The Lancet 360, 1640–1645. https://doi.org/10.1016/S0140-6736(02)11602-3

For peer review only - http://bmjopen.bmj.com/site/about/guidelines.xhtml

Power, C., Hertzman, C., 1997. Social and biological pathways linking early life and adult disease. Br. Med. Bull. 53, 210–221. https://doi.org/10.1093/oxfordjournals.bmb.a011601
Reitz, A.K., Zimmermann, J., Hutteman, R., Specht, J., Neyer, F.J., 2014. How Peers Make a Difference: The Role of Peer Groups and Peer Relationships in Personality Development: Peers and personality development. Eur. J. Personal. 28, 279–288. https://doi.org/10.1002/per.1965
Richter, M., Lampert, T., 2008. The role of socioeconomic status, peer and school context for adolescent smoking. Arch Public Health 66, 69–87.
Rose, A.J., Rudolph, K.D., 2006. A Review of Sex Differences in Peer Relationship Processes: Potential Trade-offs for the Emotional and Behavioral Development of Girls and Boys. Psychol. Bull. 132, 98–131. https://doi.org/10.1037/0033-2909.132.1.98
Rudolph, K.D., 2002. Gender differences in emotional responses to interpersonal stress during adolescence. J. Adolesc. Health 30, 3–13. https://doi.org/10.1016/S1054-139X(01)00383-4
Seeman, T.E., 1996. Social ties and health: The benefits of social integration. Ann. Epidemiol. 6, 442–451. https://doi.org/10.1016/S1047-2797(96)00095-6
Shih, J.H., Eberhart, N.K., Hammen, C.L., Brennan, P.A., 2006. Differential Exposure and Reactivity to Interpersonal Stress Predict Sex Differences in Adolescent Depression. J. Clin. Child Adolesc. Psychol. 35, 103–115. https://doi.org/10.1207/s15374424jccp3501_9
Shuey, K.M., Willson, A.E., 2014. Economic hardship in childhood and adult health trajectories: An alternative approach to investigating life-course processes. Adv. Life Course Res. 22, 49–61. https://doi.org/10.1016/j.alcr.2014.05.001
Smith, G.D., Hart, C., Blane, D., Hole, D., 1998. Adverse socioeconomic conditions in childhood and cause specific adult mortality: prospective observational study. BMJ 316, 1631–1635. https://doi.org/10.1136/bmj.316.7145.1631
Starfield, B., Riley, A., Witt, W., Robertson, J., 2002. Social class gradients in health during adolescence. J. Epidemiol. Community Health 56, 354–361. https://doi.org/10.1136/jech.56.5.354
Uchino, B.N., 2006. Social Support and Health: A Review of Physiological Processes Potentially Underlying Links to Disease Outcomes. J. Behav. Med. 29, 377–387. https://doi.org/10.1007/s10865-006-9056-5
Umberson, D., Karas Montez, J., 2010. Social Relationships and Health: A Flashpoint for Health Policy. J. Health Soc. Behav. 51, S54–S66. https://doi.org/10.1177/0022146510383501
Valtorta, N.K., Kanaan, M., Gilbody, S., Ronzi, S., Hanratty, B., 2016. Loneliness and social isolation as risk factors for coronary heart disease and stroke: systematic review and meta-analysis of longitudinal observational studies. Heart 102, 1009–1016. https://doi.org/10.1136/heartjnl-2015-308790
West, P., Sweeting, H., 2004. Evidence on equalisation in health in youth from the West of Scotland. Soc. Sci. Med. 59, 13–27. https://doi.org/10.1016/j.socscimed.2003.12.004
Wolke, D., Copeland, W.E., Angold, A., Costello, E.J., 2013. Impact of Bullying in Childhood on Adult Health, Wealth, Crime, and Social Outcomes. Psychol. Sci. 24, 1958–1970. https://doi.org/10.1177/0956797613481608
Yang, Y.C., McClintock, M.K., Kozloski, M., Li, T., 2013. Social Isolation and Adult Mortality: The Role of Chronic Inflammation and Sex Differences. J. Health Soc. Behav. 54, 183–203. https://doi.org/10.1177/0022146513485244

For peer review only - http://bmjopen.bmj.com/site/about/guidelines.xhtml

Table 1: Descriptive statistics for the study variables (n=11,400).

	Males (n=5,410)		Females (n=5,990)	
	n	%	n	%
Circulatory disease (1973-2016)				
No	4411	81.5	5321	88.8
Yes	999	18.5	669	11.2
Peer status position (1966)				
High status	1,788	33.1	1,710	28.6
Medium status	1,937	35.8	2,185	36.5
Low status	1,033	19.1	1,155	19.3
Marginalised	652	12.1	940	15.7
Sibling position (1972)				
Only child	705	13.0	705	11.8
Last born	1,714	31.7	1,929	32.2
Middle born	1,302	24.1	1,509	25.2
First born	1,689	31.2	1,847	30.8
Number of siblings (1972)				
0	705	13.0	705	11.8
1	2,090	38.6	2,245	37.5
2	1,508	27.9	1,675	28.0
3	691	12.8	815	13.6
4	254	4.7	334	5.6
More than 4	162	3.0	216	3.6
Parental educational level (1960)				
At least one parent with upper secondary degree	1,402	25.9	1,533	25.6
No parent with upper secondary degree	3,731	69.0	4,131	69.0
Missing	277	5.1	326	5.4
Parental social welfare receipt (1953-1965)				
No	4,544	84.0	5,067	84.6
Yes	866	16.0	923	15.4
Parental psychiatric problems (1953-1972)				
No	5,105	94.4	5,644	94.2
Yes	305	5.6	346	5.8
Intervention during delivery (1953)				
No	4,090	75.6	4,580	76.5
Yes	329	6.1	293	4.9
Missing	991	18.3	1,117	18.7
Cognitive ability (1966)				
1st quartile (lowest)	1,052	19.5	1,601	26.7
2nd quartile	1,268	23.4	1,538	25.7
3rd quartile	1,491	27.6	1,509	25.2
4th quartile (highest)	1,599	29.6	1,342	22.4
Conduct marks (1966)				
Not so good	5	0.1	1	0.0
Good	83	1.5	19	0.3
Very good	5,322	98.4	5,970	99.7
Average school marks (1966)				
Range 1 to 5	Mean: 3.2	Std.dev.: 0.9	Mean: 3.3	Std.dev.: 0.9
Crimes of violence (1962-1972)				
No	5,184	95.8	5,947	99.3
Yes	226	4.2	43	0.7

For peer review only - http://bmjopen.bmj.com/site/about/guidelines.xhtml

Table 2: The association between peer status position (1966) and circulatory disease (1973-2016) among males. Results from Cox proportional hazard regression models (n=5,410).

	Circulatory disease (1973-2016)									
	**Model 1**		**Model 2**		**Model 3**		**Model 4**		**Model 5**	
	**HR**	**95% CI**	**HR**	**95% CI**	**HR**	**95% CI**	**HR**	**95% CI**	**HR**	**95% CI**
Peer status position (1966)										
High status (ref.)	1		1		1		1		1	
Medium status	1.13	(0.97 - 1.32)	1.12	(0.96 - 1.31)	1.11	(0.95 - 1.29)	1.08	(0.92 - 1.26)	1.06	(0.90 - 1.24)
Low status	1.14	(0.95 - 1.37)	1.13	(0.94 - 1.36)	1.11	(0.93 - 1.33)	1.07	(0.89 - 1.28)	1.03	(0.85 - 1.24)
Marginalised	1.54***	(1.27 - 1.87)	1.53***	(1.26 - 1.86)	1.49***	(1.23 - 1.81)	1.41***	(1.16 - 1.72)	1.34**	(1.10 - 1.64)
Sibling position (1972)										
Only child (ref.)			1		1		1		1	
Last born			0.87	(0.70 - 1.09)	0.89	(0.71 - 1.11)	0.92	(0.73 - 1.15)	0.92	(0.74 - 1.16)
Middle born			0.82	(0.61 - 1.09)	0.83	(0.62 - 1.11)	0.86	(0.64 - 1.15)	0.86	(0.64 - 1.15)
First born			0.85	(0.67 - 1.06)	0.86	(0.69 - 1.09)	0.87	(0.70 - 1.10)	0.88	(0.70 - 1.11)
Number of siblings (1972)			1.06	(0.99 - 1.13)	1.04	(0.97 - 1.11)	1.03	(0.96 - 1.10)	1.03	(0.96 - 1.10)
Parental educational level (1960)										
At least one parent with upper secondary degree (ref.)					1		1		1	
No parent with upper secondary degree					1.03	(0.88 - 1.19)	0.98	(0.84 - 1.14)	0.97	(0.83 - 1.13)
Missing					1	(0.73 - 1.35)	0.9	(0.65 - 1.25)	0.9	(0.65 - 1.24)
Parental social welfare receipt (1953-1965)										
No (ref.)					1		1		1	
Yes					1.32**	(1.12 - 1.56)	1.29**	(1.08 – 1.54)	1.24*	(1.04 – 1.48)
Parental psychiatric problems (1953-1972)										
No (ref.)							1		1	
Yes							1.06	(0.81 - 1.38)	1.05	(0.81 - 1.37)
Intervention during delivery										
No (ref.)							1		1	
Yes							1.35*	(1.06 - 1.72)	1.35*	(1.06 - 1.72)
Missing							1.14	(0.96 - 1.35)	1.13	(0.95 - 1.34)
Cognitive ability (1966)										
1 quartile (lowest) (ref.)							1		1	
2nd quartile							0.9	(0.75 - 1.07)	0.91	(0.76 - 1.09)
3rd quartile							0.81*	(0.68 - 0.98)	0.83*	(0.69 - 1.00)
4th quartile (highest)							0.77**	(0.63 - 0.93)	0.79*	(0.65 - 0.95)
Conduct marks (1996)									0.65*	(0.46 - 0.92)
Average school marks (1966)									0.97	(0.90 - 1.03)
Crimes of violence (1962-1972)										
No (ref.)									1	
Yes									1.59***	(1.23 - 2.07)

HR=Hazard Ratio; CI=Confidence Interval; * = p<0.05; ** = p<0.01; *** = p<0.001

For peer review only

For peer review only - http://bmjopen.bmj.com/site/about/guidelines.xhtml

Table 3: The association between peer status position (1966) and circulatory disease (1973-2016) among females. Results from Cox proportional hazard regression models (n=5,990).

	Circulatory disease (1973-2016)									
	**Model 1**		**Model 2**		**Model 3**		**Model 4**		**Model 5**	
	**HR**	**95% CI**	**HR**	**95% CI**	**HR**	**95% CI**	**HR**	**95% CI**	**HR**	**95% CI**
Peer status position (1966)										
High status (ref.)	1		1		1		1		1	
Medium status	1.28*	(1.05 - 1.57)	1.27*	(1.03 - 1.55)	1.24*	(1.01 - 1.51)	1.20	(0.98 - 1.48)	1.18	(0.96 - 1.45)
Low status	1.42**	(1.13 - 1.79)	1.40**	(1.11 - 1.76)	1.36**	(1.08 - 1.71)	1.30*	(1.03 - 1.64)	1.26	(0.99 - 1.59)
Marginalised	1.61***	(1.27 - 2.03)	1.54***	(1.22 - 1.95)	1.49***	(1.18 - 1.89)	1.42**	(1.11 - 1.81)	1.36*	(1.07 - 1.74)
Sibling position (1972)										
Only child (ref.)			1		1		1		1	
Last born			0.76*	(0.58 - 1.00)	0.81	(0.62 - 1.07)	0.82	(0.62 - 1.07)	0.82	(0.63 - 1.08)
Middle born			0.66*	(0.47 - 0.92)	0.69*	(0.50 - 0.97)	0.68*	(0.49 - 0.96)	0.69*	(0.49 - 0.97)
First born			0.64**	(0.48 - 0.84)	0.68**	(0.51 - 0.90)	0.69**	(0.52 - 0.91)	0.69*	(0.53 - 0.92)
Number of siblings (1972)			1.12***	(1.05 - 1.19)	1.10**	(1.03 - 1.17)	1.10**	(1.03 - 1.17)	1.10**	(1.03 - 1.18)
Parental educational level (1960)										
At least one parent with upper secondary degree (ref.)					1		1		1	
No parent with upper secondary degree					1.53***	(1.25 - 1.88)	1.49***	(1.21 - 1.84)	1.48***	(1.20 - 1.82)
Missing					1.57*	(1.10 - 2.25)	1.55*	(1.05 - 2.27)	1.54*	(1.05 - 2.26)
Parental social welfare receipt (1953-1965)										
No (ref.)					1		1		1	
Yes					1.19	(0.97 - 1.45)	0.99	(0.79 - 1.24)	0.99	(0.79 - 1.23)
Parental psychiatric problems (1953-1972)										
No (ref.)							1		1	
Yes							1.74***	(1.31 - 2.30)	1.74***	(1.32 - 2.31)
Intervention during delivery										
No (ref.)							1		1	
Yes							1.26	(0.90 - 1.76)	1.25	(0.89 - 1.74)
Missing							0.98	(0.79 - 1.22)	0.98	(0.79 - 1.21)
Cognitive ability (1966)										
1 quartile (lowest) (ref.)							1		1	
2nd quartile							0.88	(0.72 - 1.08)	0.89	(0.72 - 1.09)
3rd quartile							0.95	(0.77 - 1.18)	0.97	(0.78 - 1.20)
4th quartile (highest)							0.82	(0.64 - 1.04)	0.84	(0.66 - 1.06)
Conduct marks (1996)									4.56	(0.66 - 31.66)
Average school marks (1966)									0.90*	(0.83 - 0.98)
Crimes of violence (1962-1972)										
No (ref.)									1	
Yes									2.20*	(1.16 - 4.14)

HR=Hazard Ratio; CI=Confidence Interval; * = p<0.05; ** = p<0.01; *** = p<0.001

For peer review only

Childhood peer status and circulatory disease in adulthood: a prospective cohort study

Journal:	*BMJ Open*
Manuscript ID	bmjopen-2019-036095.R1
Article Type:	Original research
Date Submitted by the Author:	19-Mar-2020
Complete List of Authors:	Miething, Alexander; Stockholms Universitet, Department of Public Health Sciences<br>Almquist, Ylva; Stockholms Universitet, Department of Public Health Sciences
**Primary Subject Heading**:	Public health
Secondary Subject Heading:	Global health
Keywords:	EPIDEMIOLOGY, PUBLIC HEALTH, SOCIAL MEDICINE

SCHOLARONE™ Manuscripts

I, the Submitting Author has the right to grant and does grant on behalf of all authors of the Work (as defined in the below author licence), an exclusive licence and/or a non-exclusive licence for contributions from authors who are: i) UK Crown employees; ii) where BMJ has agreed a CC-BY licence shall apply, and/or iii) in accordance with the terms applicable for US Federal Government officers or employees acting as part of their official duties; on a worldwide, perpetual, irrevocable, royalty-free basis to BMJ Publishing Group Ltd ("BMJ") its licensees and where the relevant Journal is co-owned by BMJ to the co-owners of the Journal, to publish the Work in this journal and any other BMJ products and to exploit all rights, as set out in our licence.

The Submitting Author accepts and understands that any supply made under these terms is made by BMJ to the Submitting Author unless you are acting as an employee on behalf of your employer or a postgraduate student of an affiliated institution which is paying any applicable article publishing charge ("APC") for Open Access articles. Where the Submitting Author wishes to make the Work available on an Open Access basis (and intends to pay the relevant APC), the terms of reuse of such Open Access shall be governed by a Creative Commons licence – details of these licences and which Creative Commons licence will apply to this Work are set out in our licence referred to above.

Other than as permitted in any relevant BMJ Author's Self Archiving Policies, I confirm this Work has not been accepted for publication elsewhere, is not being considered for publication elsewhere and does not duplicate material already published. I confirm all authors consent to publication of this Work and authorise the granting of this licence.

For peer review only

Title:

Childhood peer status and circulatory disease in adulthood: a prospective cohort study

Authors:

Alexander Miething and Ylva B Almquist

Affiliations

Department of Public Health Sciences, Stockholm University, SE-106 91, Stockholm, Sweden.

Corresponding author:

Alexander Miething

E-mail: alexander.miething@su.se

For peer review only

For peer review only - http://bmjopen.bmj.com/site/about/guidelines.xhtml

Abstract

Objectives: Childhood conditions have been recognised as important predictors of short-term and long-term health outcomes, but few studies have considered status position in the peer group as a possible determinant of adult health. Lower peer status, which often reflects experiences of marginalisation and rejection by peers, may impose inequality experiences and leave long-lasting imprints on health. The present study therefore aimed to examine whether peer status is associated with the risk for circulatory disease across adulthood.

Design: Prospective cohort study based on the Stockholm Birth Cohort Multigenerational Study (SBC Multigen).

Setting: Stockholm metropolitan area

Participants: All individuals who were born in 1953 and resident in the greater metropolitan area of Stockholm in 1963 (n=14,608). The analytical sample consisted of 5410 males and 5990 females. Peer status was sociometrically assessed when cohort members at age 13. The survey material was linked to inpatient-care registers that contained information about circulatory diseases (n=1,668) across ages 20-63. Cox proportional hazard models were used for the analysis.

Outcome measures: Circulatory disease

Results: Peer marginalisation at age 13 showed significantly higher risks of circulatory disease in adulthood among males (HR=1.34; 95% CI 1.09–1.64) and females (HR=1.33; 95% CI 1.04–1.70) alike. A graded relationship between peer status and circulatory diseases was detected in females (p=0.023). Among males, there was a threshold effect, showing that only those in the lowest status position had significantly increased risks of circulatory disease. The associations remained significant after adjusting for various conditions in child- and adulthood.

Conclusions: This study demonstrated that circulatory diseases in adulthood may be traceable to low peer status and marginalisation in childhood. It is suggested that peer status in late childhood may precede social integration in adolescence and adulthood, acting as a long-term stressor that contributes to circulatory disease through biological, behavioural, and psychosocial pathways.

For peer review only - http://bmjopen.bmj.com/site/about/guidelines.xhtml

Strengths and limitations of this study

- The longitudinal data material with linkages to register data allowed us to study the long-term implications of childhood conditions for health outcomes in adulthood.
- A relatively long follow-up period from 1973 to 2016 was considered.
- Peer status in childhood as well as other covariates were assessed at a single time point.
- Despite the use of a rich set of covariates accounting for confounding and mediation, information on certain childhood adversities (e.g., child maltreatment and peer victimisation) was unavailable.
- There was little information available about health and health behaviours in childhood and across the subsequent life course that might explain the demonstrated associations.

Keywords

Peer status; Childhood; Circulatory disease; Inpatient care; Life course; Birth cohort

For peer review only

For peer review only - http://bmjopen.bmj.com/site/about/guidelines.xhtml

Declarations

Acknowledgement

This work was supported by the Swedish Council for Health, Working Life and Social Research [grant no. 2016-07148], (http://www.forte.se/en/).

Authors' contributions

AM and YBA conceived this study. YBA prepared and compiled the data. AM performed the statistical analyses. AM and YBA wrote the manuscript. Both authors approved the final version of the manuscript.

Competing interests

The authors declare that they have no competing interests.

Availability of data and materials

The datasets generated and/or analysed during the current study are not publicly available due to ethical regulations regarding the Stockholm Birth Cohort (SBC) study but are available from the co-author on reasonable request.

Ethics approval and consent to participate

The study was approved by the Stockholm Regional Ethics Committee (reg. no. 2016/481-31/5; 2016/481-31/5). The need for consent was waived due to the retrospective nature of this study.

For peer review only

Introduction

Innumerable studies have demonstrated the importance of childhood conditions for later life outcomes and, within health inequality research, parental socioeconomic conditions are often put forward as key determinants for adult health [1–3]. For example, adverse socioeconomic circumstances in childhood have been shown to predict poor mental health, but also metabolic problems, cardiovascular diseases, and higher mortality risks [4–6]. Children nevertheless also hold positions in status structures outside the family, such as in their peer group and school contexts. Yet, relatively few studies have addressed how experiences tied to these childhood-specific types of social structure are related to health outcomes in later life [7,8]. The present study therefore aimed to investigate the long-term implications of the child's own social position – peer status – for the risk of circulatory disease, which represents one of the leading mortality causes.

Peer orientation significantly increases in early adolescence [9]. Seeking for greater autonomy, children distance themselves from their parents and simultaneously intensify their interactions with peers [10,11]. Accordingly, the role of peer- and school-related factors become increasingly important. Intensified peer orientation has also been hypothesised to have buffering effects on socioeconomic inequalities in health. In conjuncture with the "processes of equalisation" hypothesis, the socioeconomic gradient in health tends to attenuate during the child–youth transition, while experiences related to peer interactions and youth culture become more salient [12]. A plausible explanation is that peer interactions and school environment impose a homogenising effect on children's background characteristics and supersede the repercussions of children's socioeconomic origin. Instead of socioeconomic conditions, the peer context and children's position in the peer group emerge as important determinants of health and for the adoption of health behaviours. Several studies have shown that the initiation of health-related behaviours, such as smoking, is substantially driven by peers rather than determined by socioeconomic conditions [13–15].

Peers function as socialising agents and are important for children's social, emotional, and behavioural development. Interactions with peers facilitate experiences that parents cannot supply [16], and contribute to identity formation [17] and personality maturation [18]. Furthermore, these interactions have the potential to increase children's self-worth, the ability to build relationships in later life, and adaptive adjustment [19,20]. Children are usually well aware of the advantages related to higher status in the peer group [21]. Moreover, their concerns about popularity and reputation significantly increase when they approach adolescence [22]. These affection-based dimensions play an important role in the formation of sociometric peer status, which represents an established instrument to assess the degree to which child is a liked, accepted, and integrated member of the peer group [7,23].

For peer review only - http://bmjopen.bmj.com/site/about/guidelines.xhtml

Marginalisation and peer rejection – conditions that are reflected by a low peer status position – deprive children of experiences that are important for their subsequent emotional and cognitive development, and may thus increase the risk for poor well-being and reduced mental health [24]. Östberg and Modin [25] demonstrated a graded inverse relationship between peer status and long-standing illness. Accordingly, although disadvantages arise most clearly for children in the bottom group, they are also tangible for those with higher ranks in the peer hierarchy. However, it is rather obvious that marginalised and socially isolated children suffer the most as they are exceedingly vulnerable to bullying and other forms destructive peer interactions [26]. Because (pre)adolescence coincides with an increased developmental need and strong desire for peer affiliations, peer rejection and marginalisation may not only have pervasive impacts on current well-being and health, but also affect children's ability to engage in social relationship later on [21,25,27]. As acknowledged through the theory of cumulative disadvantages [28,29], adverse conditions and negative experiences in childhood tend to accrue across the life course, and magnify detriments in later health as well [30].

Among adults, stronger social integration has been linked to reduced risks of hypertension, cardiovascular diseases, obesity, and mortality [31–33], indicating that psychosocial mechanisms are at play. It is reasonable to assume that similar processes link children's peer status to later circulatory diseases [34]. Higher status positions in the peer group enable children to derive greater emotional, cognitive, and social support from social contacts and facilitate the access to tangible and non-tangible resources provided by social contacts. In line with the buffering hypothesis of social support [35], supportive social ties impose indirect protective effects on health as they enhance individuals' coping ability and moderate their neuroendocrine or behavioural responses to acute or chronic stress [36–38]. High peer status may also directly enhance individuals' self-care, achievements, and health behaviours – factors that might mediate the link to circulatory diseases in adulthood [39]. Confounding factors may nevertheless induce spurious associations between peer status and circulatory disease in later life. Plausible confounding variables are aggressive or controversial behaviours and lower cognitive abilities that are associated with peer rejection as well as poor adult health. Likewise, family-related circumstances such as number of siblings and birth order/sibling position possibly influence children's standing in the peer group, and may also impose long-term effects on health.

Based on data from a Swedish cohort born in 1953, who have been followed for more than 60 years, the present study examines the association between sociometrically assessed peer status in childhood (age 13) and circulatory disease during adulthood (ages 20-63). Drawing on earlier research that has demonstrated socioeconomic inequalities in the risks for cardiovascular and circulatory diseases, acting through psychosocial mechanisms in the adult population, an inverse association between peer status and circulatory diseases is hypothesised. In comparison to an earlier study that used the same

For peer review only - http://bmjopen.bmj.com/site/about/guidelines.xhtml

register-linked survey material [7], the present study considers a substantially longer follow-up time and a larger set of covariates to examine the role of potentially confounding and mediating factors.

Data and methods

The data material used in the current study was the Stockholm Birth Cohort Multigenerational Study (SBC Multigen). In 2018/2019, the SBC Multigen was established through a probability matching between two longitudinal, anonymised data materials. The first was the Stockholm Metropolitan Study (SMS), defined as all individuals who were born in 1953 and resident in the greater metropolitan area of Stockholm in 1963 (n=15,117). It encompasses survey and register information collected at several time points from 1953 up until 1986, after which data were anonymised. The second was RELINK53, defined as all individuals born in 1953 who lived in Sweden in 1960, 1965, and/or 1968, as well as their ascendant, contemporaneous, and descendant family members (n=2,390,753). RELINK53 contains detailed information collected from a variety of administrative registers between 1960 and 2018. An algorithm based on a set of variables present in both data materials, rendered it possible to match 14,608 of the SMS cohort members to RELINK53 (for more information, see Almquist et al [40]). Accordingly, we are able to follow these individuals from birth up until their 60s. The Regional Ethical Review Board in Stockholm approved the creation of RELINK53 as well as the probability matching to the SMS that resulted in SBC Multigen (no. 2017/34-31/5; 2017/684-32).

Patient and public involvement

The data in this study were retrospectively compiled. Therefore, patients or the public were to no time involved in the design of this study.

Dependent variable

Information on circulatory diseases was drawn from the Swedish hospital discharge register, covering the years 1973 to 2016. This register records all inpatient care events (with overnight stays) in Swedish hospitals. In this study, circulatory diseases comprised all diagnoses included in the International Classification of Diseases (ICD 10th revision; chapter IX (I00 – I99)). The diagnoses were primarily based on judgments of medical doctors. Preceding versions of the ICD (8th and 9th revision) apply to diagnoses made from 1973 to 1986, and 1987 to 1996 respectively. These older classifications were harmonised in accordance with the 10th revision of the ICD. The using variable operationalised any events of circulatory disease occurring between 1973 and 2016. If multiple events occurred, only the first was taken into account.

Explanatory variable

For peer review only - http://bmjopen.bmj.com/site/about/guidelines.xhtml

Peer status was measured through the question “Whom do you best like working with at school?” [7,41]. Students were asked to nominate three classmates, in no particular order. This sociometric test was conducted among all 6-grade students in the Stockholm metropolitan area in 1966, with the exception of school classes containing students with learning disabilities. Nominations from classmates who attended grade 6 but were not part of the cohort were also registered. Cohort members’ mean age was 12.9 years (SD=0.29). For the purpose of the current study, we additionally excluded those who attended school classes with less than 10 children, since the peer status distribution tends to be somewhat different in small classes. Based on the number of received nominations, four categories were created: “marginalised” denoted zero nominations, “low status” one nomination, “medium status” two or three nominations, and “high status” four or more nominations.

Covariates

The covariates included family-related, school-related, and individual circumstances and conditions. The family-related factors considered the presence of siblings, parents’ education and socioeconomic conditions, parents’ psychiatric problems, and conditions during delivery. Number of siblings and sibling position were assessed in 1972. The latter measured whether the study participant was the only child in the family, or whether he/she was last born, middle born, or first born. Parental educational level accounted for whether there was at least one adult in the household with an upper secondary school degree. Parents’ receipt of social welfare benefits accounts for socioeconomic difficulties and was available from the social registers, covering the period 1953 to 1965. Parental psychiatric problems (available from 1953 to 1972) were derived from the social registers and measured whether fathers or mothers showed symptoms of mental illness or psychiatric problems. Information on intervention during the delivery was based on delivery records. Cognitive ability can be regarded as individual-level confounder that affects a child’s position in the peer group but also predicts adverse health behaviours and the risk of poor health in later life. A test of cognitive ability was conducted in 1966 and measured participants’ verbal, spatial, and numerical competences. Test scores (with a possible range from 0 to 120) were grouped into quartiles of cognitive ability. Average school marks measured study participants’ school performance in the 6th grade. Average teacher-reported conduct marks assessed in the 6th grade as well as crimes of violence recorded between 1962 and 1972 indicate participants’ externalising problems. Similar to school marks, conduct marks and violent behaviour may both mediate and confound the hypothesised association between peer status and circulatory disease. Mental disorders (ICD-10; chapter V (F00 – F09; F2 – F6; F70; F99)) and disorders due to alcohol use (ICD 10; chapter V (F10)) manifested in adulthood were drawn from

For peer review only - http://bmjopen.bmj.com/site/about/guidelines.xhtml

register-based inpatient-care data (recorded from 1973 to 2016). Both measures were coded as binary variables and indicate whether a diagnosis was registered at least once between 1973 and 2016.

Study population

The study population included all cohort members in the SBC Multigen who attended school classes with at least 10 children, and had complete information on the variables of interest. Accordingly, the analytical sample resulted in 5,410 males and 5,990 females.

Statistical methods

Gender-specific cox proportional hazard models were used to estimate the risks of circulatory disease. As information on circulatory morbidity was available from 1973 to 2016, study subjects were left-truncated at 1 January 1973. Right-censoring was applied when the observation ended on 31 December 2016, or earlier, if subjects had died from any disease. Hierarchical regression was chosen as modelling strategy. Peer status was entered as single predictor in Model 1. Family-related variables were successively added to the Models 2 to 4. Model 4 additionally included individuals' cognitive ability. Model 5 also controlled for school and conduct marks, as well as crimes of violence. Finally, Model 6 further considers mental problems and disorders due to alcohol use that were diagnosed in adulthood. All analyses were performed with Stata (version 15.1).

Results

The descriptive statistics are shown in Table 1 and illustrate a higher prevalence of circulatory diseases for males (18.5 %; n=999) as compared to females (11.2 %; n=669). The distribution of peer status is relatively similar across the genders, although a slightly higher proportion of males (31.1 %; n=1,788) than females (28.6 %; n=1,710) were in high status positions, at the same time as more females (15.7 %; n=940) than males (12.1 %; n=652) experienced marginalisation.

< Table 1 about here >

The results from the Cox proportional hazard models are presented in Table 2 for males and in Table 3 for females. The unadjusted hazard ratios of males' peer status in Model 1 (Table 2) show that only those who are in a marginalised position have an increased risk for circulatory disease (HR=1.54; 95% CI 1.27–1.87). Controlling for sibling status and number of siblings in Model 2 does not affect the estimates. When adding parental educational level and social welfare receipt to the model, a small decrease of the hazard ratio for those in marginalised peer positions can be observed (Model 3). A likelihood ratio test confirmed that the decrease is significant compared to Model 2. The estimates

For peer review only - http://bmjopen.bmj.com/site/about/guidelines.xhtml

are further attenuated when we adjust for parental psychiatric problems, intervention during delivery, and cognitive ability in Model 4. Model 5 additionally considers conduct marks, average school marks, and crimes of violence. Although the hazard ratio decreases, a significant association between marginalisation and circulatory disease is retained. This association also persists when accounting for mental disorders and disorders due to alcohol misuse in Model 6.

< Table 2 about here >

Table 3 presents the corresponding analysis for females. Model 1 illustrates a graded association between peer status and circulatory disease. Females with medium peer status have a 28% higher risk of being hospitalised due to circulatory disease as compared to high status females. The respective increase by low peer status is 42%, and 61% for females in a marginalised position. As shown for males, a gradual attenuation of the hazard ratios of peer status can be observed when control variables are successively added to the model. In Model 4, the hazard ratio for medium status is no longer significant, whereas in Model 5, only marginalised females show a significant risk for circulatory disease (HR=1.36; 95% CI 1.07–1.74). A similar pattern with a somewhat lowered risk of circulatory disease in marginalised females is shown in Model 6 (HR=1.33; 95 % CI 1.04–1.70). Despite the non-significant estimates of medium and low status, a significant trend in the association between peer status and circulatory disease can still be detected (p=0.023). Whereas females' medium and low status positions indicate larger relative risks of circulatory diseases compared to males, marginalised peer status reveals remarkably similar estimates for males and females.

< Table 3 about here >

For peer review only

Discussion

The present study demonstrated that peer status in preadolescence is associated with circulatory diseases in adulthood. More specifically, being in a marginalised position in the peer group is significantly associated with increased risks of circulatory disease in both males and females. In addition, a graded association is detected for females: the lower the peer status, the higher the risk of circulatory disease. Compared to the most popular females, even those with a medium-high position in the peer group run higher risks of circulatory diseases in later life. The corresponding analyses of males revealed a threshold effect. Only socially isolated (marginalised) males displayed significantly higher hazards of circulatory disease. The demonstrated associations remained significant also after controlling for variables that could be regarded as potential confounders, including family-related factors and socioeconomic circumstances. Adjustments for school achievements (measured with school marks) and misconduct (measured with conduct marks and crimes of violence) reduced the strength of association to some, but still a non-significant extent. Mental disorders and disorders due to alcohol misuse in adulthood are likely influenced by adversities during childhood [42,43]. In line with earlier studies, these factors were demonstrated as determinants of circulatory diseases [44,45]. Therefore, the slightly lowered magnitude in the association between peer status and circulatory disease in the female sample can be surmised as a weak mediating effect of mental health and alcohol-related problems.

Altogether, our findings suggest an increased susceptibility of marginalised peers for circulatory disease in later life, which is in line with other studies that considered long-term impacts of peer integration [4,7,31,46]. However, this remains to be confirmed with data materials that also encompass additional types of confounding and mediating factors.

Past research has argued that peer status may be considered as a childhood-specific type of social structure that in many ways resemble social status positions in adult life [7,41]. Accordingly, it is plausible that lower peer status would have health implications similar to socioeconomic deprivation and low socioeconomic position in adulthood. As also outlined earlier in this study, peer status may even equalise the effects of low parental socioeconomic position [12].

The current findings contribute to research on the long-lasting impacts of childhood circumstances on health [8,47]. Studies on health implications of adults' social relationships, however, tend to disregard the emergence of socially induced health problems and the origin of destructive social experiences at earlier stages in life (Ehrlich 2015). In line with life course theory, deficient social relationships – as well as health problems – may be the result of cumulative processes that escalate with increasing age. Accordingly, earlier research conjectured a mutual interdependence of social relationships and health

For peer review only - http://bmjopen.bmj.com/site/about/guidelines.xhtml

at any stages in life course [25]. The results found in the current study suggest that this process can be backtracked to adolescence and childhood.

Among adults, stress-buffering effects of tangible and non-tangible support have been proposed as primary mechanisms as to why constructive social relationships are beneficial for health [35,39,48]. Low peer status, social isolation, and marginalisation may be particularly distressing in childhood. Socially isolated children suffer not only from lacking social and emotional support but also from the experience of having reduced opportunities to make friends and to control dominating behaviour by others [24]. Taken together, these adversities might disturb children's social and emotional development and lead to behaviours and disadvantages that are difficult to rectify in their later life. Other important aspects that potentially contribute to the findings in this study are peer victimisation and bullying. Such experiences often coincide with marginalisation and rejection. It is well documented that victimised children and youth have an increased propensity to develop somatic problems, internalising problems, anxiety and depression disorders, and tend to take up smoking [49]. Longitudinal studies have demonstrated that such problems persist throughout adulthood, which is reflected by lower mental and physical health [50,51].

Low peer status in childhood and adolescence has been identified as source of chronic (psychosocial) stress that furthers the development of mental health problems and chronic inflammation [4,52,53]. In adulthood, the lack of social contacts is associated with disorders that develop over a long time span, including metabolic problems, hypertension, cardiovascular problems, and stroke. Earlier research on childhood conditions and life course processes [54–56], as well as the findings of the current study, suggest that the childhood perspective on the one hand and adulthood perspective on the other hand, should not be considered as independent given that socially induced morbidity and mortality in adulthood may have their origin in childhood. There is convincing evidence from neuroscience regarding how social relationships modulate neuroendocrine responses that subsequently affect the circulatory system, increasing the risk for stroke and cardiovascular diseases [57]. Similar mechanisms may apply to the findings in the current study and explain why marginalisation in the peer group poses a risk factor for the development of circulatory diseases.

Another finding worth noting are the observed differences between males and females. Similar to our results, previous studies have detected a dose-response relationship between females' peer status and metabolic syndrome in adulthood [46]. The graded associations between peer status and circulatory disease in females could suggest a greater emotional and social reactivity to chronic stress in comparison to males [58,59]. Girls' greater vulnerability to interpersonal stress may further be reflected by gender differences in social interactions and acquisition of social status in the peer group.

For peer review only - http://bmjopen.bmj.com/site/about/guidelines.xhtml

Whereas girls tend to be relationship-oriented, boys are more involved in competitive and organised play [58]. Boys are also more concerned about achievements and problems directly affecting themselves [60]. These differences become manifest in girls' greater worries and self-blaming about others' relationship problems and concerns about negative evaluation by other peers. Because girls pay more attention to their peer environment and social interactions (Sneed & Cohen 2014), they may react more strongly to feedback from peers. In contrast, boys' stress pertains to school performance, physical competition, and close friendships rather than to their social networks. According to Oldehinkel (2007), likability and affection can be considered as the primary criteria of high peer status in girls, while admiration (of their achievements) marks the status position of boys. However, popular (high-status) peers are not necessarily well-liked, because peer status may also be achieved by dominance and aggression [61]. Another finding from the gender-specific analysis is the distinct association of crimes of violence with risks of circulatory diseases in males and females. The descriptive analysis clearly indicated that females' crimes of violence occur less frequent in comparison to males, which is in line with the notion that females tend to use more indirect forms of aggression [62]. The relatively high risk for circulatory disease of females involved in violent crimes may not least reflect that those females are likely to experience serious mental health problems [63] that in turn pose a risk factor for developing circulatory diseases in later life. In fact, the somewhat lower hazard ratios of females' crimes of violence after controlling for mental health problems indicate that both conditions are linked to each other.

Strengths and limitations

A particular strength of this study is the use of longitudinal data material with linkages to register data. This allowed us to study the long-term implications of childhood conditions for health outcomes in adulthood. Participation rates were high in comparison to many other studies, which reduced the effects of selection and biases from attrition and non-response. The studied single cohort covered a fairly homogeneous population. Therefore, certain spurious effects, such as influences on the presented associations due to immigration, can be ruled out. Nevertheless, this study is also subject to limitations. Peer status in childhood as well as other covariates were assessed at a single time point. Further, there was little information available about health and health behaviours in childhood and across the subsequent life course (i.e., in adolescence and adulthood) that might affect the demonstrated associations. For example, possible confounding effects of child maltreatment and peer victimisation, or mediating effects of smoking and substance abuse, could not be addressed in the present study. Moreover, it was not possible to determine when health problems (e.g., the predecessors of circulatory diseases) emerged. Likewise, potentially relevant information about circumstances in adulthood (e.g. achievements and ambitions) was not available in the data. It was

For peer review only - http://bmjopen.bmj.com/site/about/guidelines.xhtml

For peer review only

therefore not possible to fully identify the causal pathways, for instance, whether psychological, behavioural, or biological processes contributed to demonstrated associations between peer status and circulatory disease. Despite the use of controls for parental socioeconomic status and other confounders such as family circumstances, it is nevertheless possible that selection and unmeasured confounding affected the identified associations. Given these limitations, we cannot draw any causal inference from this study.

Conclusions

This study confirmed that childhood circumstances impose health effects in later life. Peer relations do not only play an important role for children's emotional and social development but may have considerable long-term implications on their health. Congruent with earlier studies on the role of socioeconomic conditions for morbidity and mortality, the findings of this study stress the importance of children's social relationships for circulatory diseases. Our investigation of this rather specific outcome might help to understand the processes that contribute to socially induced morbidity and mortality.

For peer review only - http://bmjopen.bmj.com/site/about/guidelines.xhtml

References

1 Cohen S, Janicki-Deverts D, Chen E, *et al.* Childhood socioeconomic status and adult health: Childhood socioeconomic status and adult health. *Ann N Y Acad Sci* 2010;**1186**:37–55. doi:10.1111/j.1749-6632.2009.05334.x

2 Galobardes B. Childhood Socioeconomic Circumstances and Cause-specific Mortality in Adulthood: Systematic Review and Interpretation. *Epidemiol Rev* 2004;**26**:7–21. doi:10.1093/epirev/mxh008

3 Poulton R, Caspi A, Milne BJ, *et al.* Association between children's experience of socioeconomic disadvantage and adult health: a life-course study. *The Lancet* 2002;**360**:1640–5. doi:10.1016/S0140-6736(02)11602-3

4 Danese A, Moffitt TE, Harrington H, *et al.* Adverse Childhood Experiences and Adult Risk Factors for Age-Related Disease: Depression, Inflammation, and Clustering of Metabolic Risk Markers. *Arch Pediatr Adolesc Med* 2009;**163**. doi:10.1001/archpediatrics.2009.214

5 Galobardes B, Smith GD, Lynch JW. Systematic Review of the Influence of Childhood Socioeconomic Circumstances on Risk for Cardiovascular Disease in Adulthood. *Ann Epidemiol* 2006;**16**:91–104. doi:10.1016/j.annepidem.2005.06.053

6 Smith GD, Hart C, Blane D, *et al.* Adverse socioeconomic conditions in childhood and cause specific adult mortality: prospective observational study. *BMJ* 1998;**316**:1631–5. doi:10.1136/bmj.316.7145.1631

7 Almquist Y. Peer status in school and adult disease risk: a 30-year follow-up study of disease-specific morbidity in a Stockholm cohort. *J Epidemiol Community Health* 2009;**63**:1028–34. doi:10.1136/jech.2009.088377

8 Caspi A, Harrington H, Moffitt TE, *et al.* Socially Isolated Children 20 Years Later: Risk of Cardiovascular Disease. *Arch Pediatr Adolesc Med* 2006;**160**:805. doi:10.1001/archpedi.160.8.805

9 Fuligni AJ, Eccles JS, Barber BL, *et al.* Early adolescent peer orientation and adjustment during high school. *Dev Psychol* 2001;**37**:28–36. doi:10.1037//0012-1649.37.1.28

10 Fuligni AJ, Eccles JS. Perceived parent-child relationships and early adolescents' orientation toward peers. *Dev Psychol* 1993;**29**:622–32. doi:10.1037/0012-1649.29.4.622

11 Starfield B, Riley A, Witt W, *et al.* Social class gradients in health during adolescence. *J Epidemiol Community Health* 2002;**56**:354–61. doi:10.1136/jech.56.5.354

12 West P, Sweeting H. Evidence on equalisation in health in youth from the West of Scotland. *Soc Sci Med* 2004;**59**:13–27. doi:10.1016/j.socscimed.2003.12.004

13 Hanson MD, Chen E. Socioeconomic Status and Health Behaviors in Adolescence: A Review of the Literature. *J Behav Med* 2007;**30**:263–85. doi:10.1007/s10865-007-9098-3

14 Kobus K. Peers and adolescent smoking. *Addiction* 2003;**98**:37–55. doi:10.1046/j.1360-0443.98.s1.4.x

For peer review only - http://bmjopen.bmj.com/site/about/guidelines.xhtml

15 Richter M, Lampert T. The role of socioeconomic status, peer and school context for adolescent smoking. *Arch Public Health* 2008;**66**:69–87.

16 Bagwell CL, Newcomb AF, Bukowski WM. Preadolescent Friendship and Peer Rejection as Predictors of Adult Adjustment. *Child Dev* 1998;**69**:140–53. doi:10.1111/j.1467-8624.1998.tb06139.x

17 Brown BB, Eicher SA, Petrie S. The importance of peer group ("crowd") affiliation in adolescence. *J Adolesc* 1986;**9**:73–96. doi:10.1016/S0140-1971(86)80029-X

18 Reitz AK, Zimmermann J, Hutteman R, *et al.* How Peers Make a Difference: The Role of Peer Groups and Peer Relationships in Personality Development: Peers and personality development. *Eur J Personal* 2014;**28**:279–88. doi:10.1002/per.1965

19 LaFontana KM, Cillessen AHN. Children's perceptions of popular and unpopular peers: A multimethod assessment. *Dev Psychol* 2002;**38**:635–47. doi:10.1037//0012-1649.38.5.635

20 Parker JG, Asher SR. Peer relations and later personal adjustment: Are low-accepted children at risk? *Psychol Bull* 1987;**102**:357–89. doi:10.1037/0033-2909.102.3.357

21 Lease AM, Musgrove KT, Axelrod JL. Dimensions of Social Status in Preadolescent Peer Groups: Likability, Perceived Popularity, and Social Dominance. *Soc Dev* 2002;**11**:508–33. doi:10.1111/1467-9507.00213

22 Fournier MA. Adolescent Hierarchy Formation and the Social Competition Theory of Depression. *J Soc Clin Psychol* 2009;**28**:1144–72. doi:10.1521/jscp.2009.28.9.1144

23 Parkhurst JT, Hopmeyer A. Sociometric Popularity and Peer-Perceived Popularity: Two Distinct Dimensions of Peer Status. *J Early Adolesc* 1998;**18**:125–44. doi:10.1177/0272431698018002001

24 Oldehinkel AJ, Rosmalen JGM, Veenstra R, *et al.* Being Admired or Being Liked: Classroom Social Status and Depressive Problems in Early Adolescent Girls and Boys. *J Abnorm Child Psychol* 2007;**35**:417–27. doi:10.1007/s10802-007-9100-0

25 Östberg V, Modin B. Status relations in school and their relevance for health in a life course perspective: Findings from the Aberdeen children of the 1950's cohort study. *Soc Sci Med* 2008;**66**:835–48. doi:10.1016/j.socscimed.2007.10.018

26 Wolke D, Copeland WE, Angold A, *et al.* Impact of Bullying in Childhood on Adult Health, Wealth, Crime, and Social Outcomes. *Psychol Sci* 2013;**24**:1958–70. doi:10.1177/0956797613481608

27 Plenty S, Jonsson JO. Social Exclusion among Peers: The Role of Immigrant Status and Classroom Immigrant Density. *J Youth Adolesc* 2017;**46**:1275–88. doi:10.1007/s10964-016-0564-5

28 Dannefer D. Cumulative Advantage/Disadvantage and the Life Course: Cross-Fertilizing Age and Social Science Theory. *J Gerontol B Psychol Sci Soc Sci* 2003;**58**:S327–37. doi:10.1093/geronb/58.6.S327

29 DiPrete TA, Eirich GM. Cumulative Advantage as a Mechanism for Inequality: A Review of Theoretical and Empirical Developments. *Annu Rev Sociol* 2006;**32**:271–97. doi:10.1146/annurev.soc.32.061604.123127

For peer review only - http://bmjopen.bmj.com/site/about/guidelines.xhtml

30 Shuey KM, Willson AE. Economic hardship in childhood and adult health trajectories: An alternative approach to investigating life-course processes. *Adv Life Course Res* 2014;**22**:49–61. doi:10.1016/j.alcr.2014.05.001

31 Cundiff JM, Matthews KA. Friends With Health Benefits: The Long-Term Benefits of Early Peer Social Integration for Blood Pressure and Obesity in Midlife. *Psychol Sci* 2018;**29**:814–23. doi:10.1177/0956797617746510

32 House JS, Landis KR, Umberson D. Social Relationships and Health. *Science* 1988;**241**:540.

33 Valtorta NK, Kanaan M, Gilbody S, *et al.* Loneliness and social isolation as risk factors for coronary heart disease and stroke: systematic review and meta-analysis of longitudinal observational studies. *Heart* 2016;**102**:1009–16. doi:10.1136/heartjnl-2015-308790

34 Kroenke C. Socioeconomic status and health: Youth development and neomaterialist and psychosocial mechanisms. *Soc Sci Med* 2008;**66**:31–42. doi:10.1016/j.socscimed.2007.07.018

35 Cohen S, Wills TA. Stress, social support, and the buffering hypothesis. *Psychol Bull* 1985;**98**:310–57. doi:10.1037/0033-2909.98.2.310

36 Holt-Lunstad J, Smith TB, Layton JB. Social Relationships and Mortality Risk: A Meta-analytic Review. *PLoS Med* 2010;**7**:e1000316. doi:10.1371/journal.pmed.1000316

37 Seeman TE. Social ties and health: The benefits of social integration. *Ann Epidemiol* 1996;**6**:442–51. doi:10.1016/S1047-2797(96)00095-6

38 Umberson D, Karas Montez J. Social Relationships and Health: A Flashpoint for Health Policy. *J Health Soc Behav* 2010;**51**:S54–66. doi:10.1177/0022146510383501

39 Uchino BN. Social Support and Health: A Review of Physiological Processes Potentially Underlying Links to Disease Outcomes. *J Behav Med* 2006;**29**:377–87. doi:10.1007/s10865-006-9056-5

40 Almquist YB, Grotta A, Vågerö D, *et al.* Cohort profile update: The Stockholm birth cohort study. *Int J Epidemiol* Published Online First: 20 September 2019. doi:10.1093/ije/dyz185

41 Almquist Y. Social isolation in the classroom and adult health: A longitudinal study of a 1953 cohort. *Adv Life Course Res* 2011;**16**:1–12. doi:10.1016/j.alcr.2010.11.001

42 Hughes K, Bellis MA, Hardcastle KA, *et al.* The effect of multiple adverse childhood experiences on health: a systematic review and meta-analysis. *Lancet Public Health* 2017;**2**:e356–66. doi:10.1016/S2468-2667(17)30118-4

43 Shin SH, McDonald SE, Conley D. Patterns of adverse childhood experiences and substance use among young adults: A latent class analysis. *Addict Behav* 2018;**78**:187–92. doi:10.1016/j.addbeh.2017.11.020

44 Glaus J, Vandeleur C, Gholam-Rezaee M, *et al.* Atypical depression and alcohol misuse are related to the cardiovascular risk in the general population. *Acta Psychiatr Scand* 2012;:n/a-n/a. doi:10.1111/acps.12057

45 Goodwin RD, Davidson KW, Keyes K. Mental disorders and cardiovascular disease among adults in the United States. *J Psychiatr Res* 2009;**43**:239–46. doi:10.1016/j.jpsychires.2008.05.006

For peer review only - http://bmjopen.bmj.com/site/about/guidelines.xhtml

46 Gustafsson PE, Janlert U, Theorell T, *et al.* Do Peer Relations in Adolescence Influence Health in Adulthood? Peer Problems in the School Setting and the Metabolic Syndrome in Middle-Age. *PLoS ONE* 2012;**7**:e39385. doi:10.1371/journal.pone.0039385

47 Shonkoff JP, Garner AS, THE COMMITTEE ON PSYCHOSOCIAL ASPECTS OF CHILD AND FAMILY HEALTH, COMMITTEE ON EARLY CHILDHOOD, ADOPTION, AND DEPENDENT CARE, AND SECTION ON DEVELOPMENTAL AND BEHAVIORAL PEDIATRICS, *et al.* The Lifelong Effects of Early Childhood Adversity and Toxic Stress. *PEDIATRICS* 2012;**129**:e232–46. doi:10.1542/peds.2011-2663

48 Rueger SY, Malecki CK, Pyun Y, *et al.* A meta-analytic review of the association between perceived social support and depression in childhood and adolescence. *Psychol Bull* 2016;**142**:1017–67. doi:10.1037/bul0000058

49 Wolke D, Lereya ST. Long-term effects of bullying. *Arch Dis Child* 2015;**100**:879–85. doi:10.1136/archdischild-2014-306667

50 Copeland WE, Wolke D, Lereya ST, *et al.* Childhood bullying involvement predicts low-grade systemic inflammation into adulthood. *Proc Natl Acad Sci* 2014;**111**:7570–5. doi:10.1073/pnas.1323641111

51 Lereya ST, Copeland WE, Costello EJ, *et al.* Adult mental health consequences of peer bullying and maltreatment in childhood: two cohorts in two countries. *Lancet Psychiatry* 2015;**2**:524–31. doi:10.1016/S2215-0366(15)00165-0

52 Nurius PS, Green S, Logan-Greene P, *et al.* Life course pathways of adverse childhood experiences toward adult psychological well-being: A stress process analysis. *Child Abuse Negl* 2015;**45**:143–53. doi:10.1016/j.chiabu.2015.03.008

53 Yang YC, McClintock MK, Kozloski M, *et al.* Social Isolation and Adult Mortality: The Role of Chronic Inflammation and Sex Differences. *J Health Soc Behav* 2013;**54**:183–203. doi:10.1177/0022146513485244

54 Hayward MD, Gorman BK. The Long Arm of Childhood: The Influence of Early-Life Social Conditions on Men's Mortality. *Demography* 2004;**41**:87–107. doi:10.1353/dem.2004.0005

55 Miller GE, Chen E, Parker KJ. Psychological stress in childhood and susceptibility to the chronic diseases of aging: Moving toward a model of behavioral and biological mechanisms. *Psychol Bull* 2011;**137**:959–97. doi:10.1037/a0024768

56 Power C, Hertzman C. Social and biological pathways linking early life and adult disease. *Br Med Bull* 1997;**53**:210–21. doi:10.1093/oxfordjournals.bmb.a011601

57 Booth J, Connelly L, Lawrence M, *et al.* Evidence of perceived psychosocial stress as a risk factor for stroke in adults: a meta-analysis. *BMC Neurol* 2015;**15**. doi:10.1186/s12883-015-0456-4

58 Rose AJ, Rudolph KD. A Review of Sex Differences in Peer Relationship Processes: Potential Trade-offs for the Emotional and Behavioral Development of Girls and Boys. *Psychol Bull* 2006;**132**:98–131. doi:10.1037/0033-2909.132.1.98

59 Rudolph KD. Gender differences in emotional responses to interpersonal stress during adolescence. *J Adolesc Health* 2002;**30**:3–13. doi:10.1016/S1054-139X(01)00383-4

For peer review only - http://bmjopen.bmj.com/site/about/guidelines.xhtml

60 Shih JH, Eberhart NK, Hammen CL, *et al.* Differential Exposure and Reactivity to Interpersonal Stress Predict Sex Differences in Adolescent Depression. *J Clin Child Adolesc Psychol* 2006;**35**:103–15. doi:10.1207/s15374424jccp3501_9

61 Vaillancourt T, Hymel S. Aggression and social status: the moderating roles of sex and peer-valued characteristics. *Aggress Behav* 2006;**32**:396–408. doi:10.1002/ab.20138

62 Salmivalli C, Kaukiainen A. “Female aggression” revisited: Variable- and person-centered approaches to studying gender differences in different types of aggression. *Aggress Behav* 2004;**30**:158–63. doi:10.1002/ab.20012

63 Cauffman E, Lexcen FJ, Goldweber A, *et al.* Gender Differences in Mental Health Symptoms Among Delinquent and Community Youth. *Youth Violence Juv Justice* 2007;**5**:287–307. doi:10.1177/1541204007301292

For peer review only

Table 1: Descriptive statistics for the study variables (n=11,400).

	Males (n=5,410)		Females (n=5,990)	
	n	%	n	%
Circulatory disease (1973-2016)				
No	4,411	81.5	5,321	88.8
Yes	999	18.5	669	11.2
Peer status position (1966)				
High status	1,788	33.1	1,710	28.6
Medium status	1,937	35.8	2,185	36.5
Low status	1,033	19.1	1,155	19.3
Marginalised	652	12.1	940	15.7
Sibling position (1972)				
Only child	705	13.0	705	11.8
Last born	1,714	31.7	1,929	32.2
Middle born	1,302	24.1	1,509	25.2
First born	1,689	31.2	1,847	30.8
Number of siblings (1972)				
0	705	13.0	705	11.8
1	2,090	38.6	2,245	37.5
2	1,508	27.9	1,675	28.0
3	691	12.8	815	13.6
4	254	4.7	334	5.6
More than 4	162	3.0	216	3.6
Parental educational level (1960)				
At least one parent with upper secondary degree	1,402	25.9	1,533	25.6
No parent with upper secondary degree	3,731	69.0	4,131	69.0
Missing	277	5.1	326	5.4
Parental social welfare receipt (1953-1965)				
No	4,544	84.0	5,067	84.6
Yes	866	16.0	923	15.4
Parental psychiatric problems (1953-1972)				
No	5,105	94.4	5,644	94.2
Yes	305	5.6	346	5.8
Intervention during delivery (1953)				
No	4,090	75.6	4,580	76.5
Yes	329	6.1	293	4.9
Missing	991	18.3	1,117	18.7
Cognitive ability (1966)				
1st quartile (lowest)	1,052	19.5	1,601	26.7
2nd quartile	1,268	23.4	1,538	25.7
3rd quartile	1,491	27.6	1,509	25.2
4th quartile (highest)	1,599	29.6	1,342	22.4
Conduct marks (1966)				
Not so good	5	0.1	1	0.0
Good	83	1.5	19	0.3
Very good	5,322	98.4	5,970	99.7
Average school marks (1966)				
Range 1 to 5	Mean: 3.2	Std.dev.: 0.9	Mean: 3.3	Std.dev.: 0.9
Crimes of violence (1962-1972)				
No	5,184	95.8	5,947	99.3
Yes	226	4.2	43	0.7
Mental disorders (1973-2016)				
No	5,051	93.4	5,535	92.4
Yes	359	6.6	455	7.6
Disorders due to alcohol use (1973-2016)				
No	5,070	93.7	5,804	96.9
Yes	340	6.3	186	3.1

For peer review only

For peer review only - http://bmjopen.bmj.com/site/about/guidelines.xhtml

Table 2: The association between peer status position (1966) and circulatory disease (1973-2016) among males. Results from Cox proportional hazard regression models (n=5,410).

	Circulatory disease (1973-2016)											
	**Model 1**		**Model 2**		**Model 3**		**Model 4**		**Model 5**		**Model 6**	
	**HR**	**95% CI**	**HR**	**95% CI**	**HR**	**95% CI**	**HR**	**95% CI**	**HR**	**95% CI**	**HR**	**95% CI**
Peer status position (1966)												
High status (ref.)	1		1		1		1		1		1	
Medium status	1.13	(0.97 - 1.32)	1.12	(0.96 - 1.31)	1.11	(0.95 - 1.29)	1.08	(0.92 - 1.26)	1.06	(0.90 - 1.24)	1.05	(0.89 - 1.23)
Low status	1.14	(0.95 - 1.37)	1.13	(0.94 - 1.36)	1.11	(0.93 - 1.33)	1.07	(0.89 - 1.28)	1.03	(0.85 - 1.24)	1.01	(0.84 - 1.22)
Marginalised	1.54***	(1.27 - 1.87)	1.53***	(1.26 - 1.86)	1.49***	(1.23 - 1.81)	1.41***	(1.16 - 1.72)	1.34**	(1.10 - 1.64)	1.34**	(1.09 - 1.64)
Sibling position (1972)												
Only child (ref.)			1		1		1		1		1	
Last born			0.87	(0.70 - 1.09)	0.89	(0.71 - 1.11)	0.92	(0.73 - 1.15)	0.92	(0.74 - 1.16)	0.93	(0.74 - 1.17)
Middle born			0.82	(0.61 - 1.09)	0.83	(0.62 - 1.11)	0.86	(0.64 - 1.15)	0.86	(0.64 - 1.15)	0.88	(0.66 - 1.18)
First born			0.85	(0.67 - 1.06)	0.86	(0.69 - 1.09)	0.87	(0.70 - 1.10)	0.88	(0.70 - 1.11)	0.89	(0.71 - 1.12)
Number of siblings (1972)			1.06	(0.99 - 1.13)	1.04	(0.97 - 1.11)	1.03	(0.96 - 1.10)	1.03	(0.96 - 1.10)	1.02	(0.95 - 1.09)
Parental educational level (1960)												
At least one parent with upper secondary degree (ref.)					1		1		1		1	
No parent with upper secondary degree					1.03	(0.88 - 1.19)	0.98	(0.84 - 1.14)	0.97	(0.83 - 1.13)	0.97	(0.83 - 1.14)
Missing					1.00	(0.73 - 1.35)	0.90	(0.65 - 1.25)	0.90	(0.65 - 1.24)	0.91	(0.66 - 1.25)
Parental social welfare receipt (1953-1965)												
No (ref.)					1		1		1		1	
Yes					1.32**	(1.12 - 1.56)	1.29**	(1.08 - 1.54)	1.24*	(1.04 - 1.48)	1.21*	(1.01 - 1.45)
Parental psychiatric problems (1953-1972)												
No (ref.)							1		1		1	
Yes							1.06	(0.81 - 1.38)	1.05	(0.81 - 1.37)	1.00	(0.77 - 1.31)
Intervention during delivery												
No (ref.)							1		1		1	
Yes							1.35*	(1.06 - 1.72)	1.35*	(1.06 - 1.72)	1.35*	(1.06 - 1.72)
Missing							1.14	(0.96 - 1.35)	1.13	(0.95 - 1.34)	1.13	(0.95 - 1.34)
Cognitive ability (1966)												
1st quartile (lowest) (ref.)							1		1		1	
2nd quartile							0.90	(0.75 - 1.07)	0.91	(0.76 - 1.09)	0.94	(0.78 - 1.13)
3rd quartile							0.81*	(0.68 - 0.98)	0.83*	(0.69 - 1.00)	0.86	(0.72 - 1.04)
4th quartile (highest)							0.77**	(0.63 - 0.93)	0.79*	(0.65 - 0.95)	0.82*	(0.68 - 1.00)
Conduct marks (1966)									0.65*	(0.46 - 0.92)	0.68*	(0.48 - 0.96)
Average school marks (1966)									0.97	(0.90 - 1.03)	0.96	(0.90 - 1.03)
Crimes of violence (1962-1972)												
No (ref.)									1		1	
Yes									1.59**	(1.23 - 2.07)	1.44**	(1.10 - 1.87)
Mental disorders (1973-2016)												
No (ref.)											1	
Yes											1.43**	(1.14 - 1.79)
Disorders due to alcohol use (1973-2016)												
No (ref.)											1	
Yes											1.74***	(1.39 - 2.17)

HR=Hazard Ratio; CI=Confidence Interval; * = p<0.05; ** = p<0.01; *** = p<0.001

For peer review only

For peer review only - http://bmjopen.bmj.com/site/about/guidelines.xhtml

Table 3: The association between peer status position (1966) and circulatory disease (1973-2016) among females. Results from Cox proportional hazard regression models (n=5,990).

	Circulatory disease (1973-2016)											
	Model 1		Model 2		Model 3		Model 4		Model 5		Model 6	
	HR	95% CI	HR	95% CI	HR	95% CI	HR	95% CI	HR	95% CI	HR	95% CI
Peer status position (1966)												
High status (ref.)	1		1		1		1		1		1	
Medium status	1.28*	(1.05 - 1.57)	1.27*	(1.03 - 1.55)	1.24*	(1.01 - 1.51)	1.20	(0.98 - 1.48)	1.18	(0.96 - 1.45)	1.18	(0.96 - 1.45)
Low status	1.42**	(1.13 - 1.79)	1.40**	(1.11 - 1.76)	1.36**	(1.08 - 1.71)	1.30*	(1.03 - 1.64)	1.26	(0.99 - 1.59)	1.24	(0.98 - 1.57)
Marginalised	1.61***	(1.27 - 2.03)	1.54***	(1.22 - 1.95)	1.49***	(1.18 - 1.89)	1.42**	(1.11 - 1.81)	1.36*	(1.07 - 1.74)	1.33*	(1.04 - 1.70)
Sibling position (1972)												
Only child (ref.)			1		1		1		1		1	
Last born			0.76*	(0.58 - 1.00)	0.81	(0.62 - 1.07)	0.82	(0.62 - 1.07)	0.82	(0.63 - 1.08)	0.83	(0.63 - 1.10)
Middle born			0.66*	(0.47 - 0.92)	0.69*	(0.50 - 0.97)	0.68*	(0.49 - 0.96)	0.69*	(0.49 - 0.97)	0.70*	(0.50 - 0.98)
First born			0.64**	(0.48 - 0.84)	0.68**	(0.51 - 0.90)	0.69**	(0.52 - 0.91)	0.69*	(0.53 - 0.92)	0.70*	(0.53 - 0.93)
Number of siblings (1972)			1.12***	(1.05 - 1.19)	1.10**	(1.03 - 1.17)	1.10**	(1.03 - 1.17)	1.10**	(1.03 - 1.18)	1.10**	(1.03 - 1.18)
Parental educational level (1960)												
At least one parent with upper secondary degree (ref.)					1		1		1		1	
No parent with upper secondary degree					1.53***	(1.25 - 1.88)	1.49***	(1.21 - 1.84)	1.48***	(1.20 - 1.82)	1.48**	(1.20 - 1.82)
Missing					1.57*	(1.10 - 2.25)	1.55*	(1.05 - 2.27)	1.54*	(1.05 - 2.26)	1.52*	(1.03 - 2.22)
Parental social welfare receipt (1953-1965)												
No (ref.)					1		1		1		1	
Yes					1.19	(0.97 - 1.45)	0.99	(0.79 - 1.24)	0.99	(0.79 - 1.23)	0.97	(0.77 - 1.21)
Parental psychiatric problems (1953-1972)												
No (ref.)							1		1		1	
Yes							1.74***	(1.31 - 2.30)	1.74***	(1.32 - 2.31)	1.68**	(1.27 - 2.23)
Intervention during delivery												
No (ref.)							1		1		1	
Yes							1.26	(0.90 - 1.76)	1.25	(0.89 - 1.74)	1.24	(0.89 - 1.73)
Missing							0.98	(0.79 - 1.22)	0.98	(0.79 - 1.21)	0.97	(0.78 - 1.21)
Cognitive ability (1966)												
1st quartile (lowest) (ref.)							1		1		1	
2nd quartile							0.88	(0.72 - 1.08)	0.89	(0.72 - 1.09)	0.91	(0.74 - 1.12)
3rd quartile							0.95	(0.77 - 1.18)	0.97	(0.78 - 1.20)	1.00	(0.81 - 1.24)
4th quartile (highest)							0.82	(0.64 - 1.04)	0.84	(0.66 - 1.06)	0.87	(0.68 - 1.11)
Conduct marks (1966)									4.56	(0.66 - 31.66)	4.01	(0.58 - 27.70)
Average school marks (1966)									0.90*	(0.83 - 0.98)	0.90*	(0.83 - 0.98)
Crimes of violence (1962-1972)												
No (ref.)									1		1	
Yes									2.20*	(1.16 - 4.14)	1.86	(0.98 - 3.53)
Mental disorders (1973-2016)												
No (ref.)											1	
Yes											1.38*	(1.07 - 1.77)
Disorders due to alcohol use (1973-2016)												
No (ref.)											1	
Yes											1.94***	(1.40 - 2.69)

HR=Hazard Ratio; CI=Confidence Interval; * = p<0.05; ** = p<0.01; *** = p<0.001

For peer review only

For peer review only - http://bmjopen.bmj.com/site/about/guidelines.xhtml

STROBE 2007 (v4) Statement—Checklist of items that should be included in reports of *cohort studies*

Section/Topic	Item #	Recommendation	Reported on page #
**Title and abstract**	1	(*a*) Indicate the study's design with a commonly used term in the title or the abstract	2 (line 8)
		(*b*) Provide in the abstract an informative and balanced summary of what was done and what was found	2 (lines 23 – 26)
**Introduction**			
Background/rationale	2	Explain the scientific background and rationale for the investigation being reported	5 (lines 2 – 24)
Objectives	3	State specific objectives, including any prespecified hypotheses	6 (lines 31 – 34)
**Methods**			
Study design	4	Present key elements of study design early in the paper	2 (lines 11 – 15)
Setting	5	Describe the setting, locations, and relevant dates, including periods of recruitment, exposure, follow-up, and data collection	7 (lines 5 – 16)
Participants	6	(*a*) Give the eligibility criteria, and the sources and methods of selection of participants. Describe methods of follow-up	7 (lines 6 – 15); 9 (lines 4 – 6)
		(*b*) For matched studies, give matching criteria and number of exposed and unexposed	7 (lines 5 – 16)
Variables	7	Clearly define all outcomes, exposures, predictors, potential confounders, and effect modifiers. Give diagnostic criteria, if applicable	7 (lines 23 – 31); 8 (lines 1 – 33)
Data sources/ measurement	8*	For each variable of interest, give sources of data and details of methods of assessment (measurement). Describe comparability of assessment methods if there is more than one group	7 (line 8/9); 8
Bias	9	Describe any efforts to address potential sources of bias	8 (lines 13 – 33)
Study size	10	Explain how the study size was arrived at	7 (lines 5 – 16); 9 (lines 9 – 11)
Quantitative variables	11	Explain how quantitative variables were handled in the analyses. If applicable, describe which groupings were chosen and why	9 (lines 11 – 15)
Statistical methods	12	(*a*) Describe all statistical methods, including those used to control for confounding	9 (lines 7 – 16)
		(*b*) Describe any methods used to examine subgroups and interactions	Not applicable
		(*c*) Explain how missing data were addressed	9 (lines 4, 5)
		(*d*) If applicable, explain how loss to follow-up was addressed	Not applicable
		(*e*) Describe any sensitivity analyses	Not applicable

For peer review only

**Results**			
Participants	13*	(a) Report numbers of individuals at each stage of study—eg numbers potentially eligible, examined for eligibility, confirmed eligible, included in the study, completing follow-up, and analysed	7 (lines 7 – 11); 8 (lines 2 – 4); Table 1
		(b) Give reasons for non-participation at each stage	7 (lines 7 – 12); 8 (lines 2 – 4)
		(c) Consider use of a flow diagram	Not applicable
Descriptive data	14*	(a) Give characteristics of study participants (eg demographic, clinical, social) and information on exposures and potential confounders	7 (lines 5 – 17); 8; 9 (lines 1 – 16); Table 1
		(b) Indicate number of participants with missing data for each variable of interest	Table 1
		(c) Summarise follow-up time (eg, average and total amount)	9 (lines 9 – 11)
Outcome data	15*	Report numbers of outcome events or summary measures over time	9 (line 20)
Main results	16	(*a*) Give unadjusted estimates and, if applicable, confounder-adjusted estimates and their precision (eg, 95% confidence interval). Make clear which confounders were adjusted for and why they were included	9 (lines 11 – 15); Table 2 and 3
		(*b*) Report category boundaries when continuous variables were categorized	8 (line 27)
		(*c*) If relevant, consider translating estimates of relative risk into absolute risk for a meaningful time period	Not applicable
Other analyses	17	Report other analyses done—eg analyses of subgroups and interactions, and sensitivity analyses	Not applicable
**Discussion**			
Key results	18	Summarise key results with reference to study objectives	14 (lines 8 – 12)
**Limitations**			
Interpretation	20	Give a cautious overall interpretation of results considering objectives, limitations, multiplicity of analyses, results from similar studies, and other relevant evidence	11 (lines 2 – 18); 12; 13
Generalisability	21	Discuss the generalisability (external validity) of the study results	13 (lines 21 – 26)
**Other information**			
Funding	22	Give the source of funding and the role of the funders for the present study and, if applicable, for the original study on which the present article is based	4 (lines 3, 4)

*Give information separately for cases and controls in case-control studies and, if applicable, for exposed and unexposed groups in cohort and cross-sectional studies.

For peer review only

Note: An Explanation and Elaboration article discusses each checklist item and gives methodological background and published examples of transparent reporting. The STROBE checklist is best used in conjunction with this article (freely available on the Web sites of PLoS Medicine at http://www.plosmedicine.org/, Annals of Internal Medicine at http://www.annals.org/, and Epidemiology at http://www.epidem.com/). Information on the STROBE Initiative is available at www.strobe-statement.org.

For peer review only

BMJ Open

Childhood peer status and circulatory disease in adulthood: a prospective cohort study in Stockholm, Sweden

Journal:	*BMJ Open*
Manuscript ID	bmjopen-2019-036095.R2
Article Type:	Original research
Date Submitted by the Author:	07-Apr-2020
Complete List of Authors:	Miething, Alexander; Stockholms Universitet, Department of Public Health Sciences<br>Almquist, Ylva; Stockholms Universitet, Department of Public Health Sciences
**Primary Subject Heading**:	Public health
Secondary Subject Heading:	Global health
Keywords:	EPIDEMIOLOGY, PUBLIC HEALTH, SOCIAL MEDICINE

SCHOLARONE™
Manuscripts

I, the Submitting Author has the right to grant and does grant on behalf of all authors of the Work (as defined in the below author licence), an exclusive licence and/or a non-exclusive licence for contributions from authors who are: i) UK Crown employees; ii) where BMJ has agreed a CC-BY licence shall apply, and/or iii) in accordance with the terms applicable for US Federal Government officers or employees acting as part of their official duties; on a worldwide, perpetual, irrevocable, royalty-free basis to BMJ Publishing Group Ltd ("BMJ") its licensees and where the relevant Journal is co-owned by BMJ to the co-owners of the Journal, to publish the Work in this journal and any other BMJ products and to exploit all rights, as set out in our licence.

The Submitting Author accepts and understands that any supply made under these terms is made by BMJ to the Submitting Author unless you are acting as an employee on behalf of your employer or a postgraduate student of an affiliated institution which is paying any applicable article publishing charge ("APC") for Open Access articles. Where the Submitting Author wishes to make the Work available on an Open Access basis (and intends to pay the relevant APC), the terms of reuse of such Open Access shall be governed by a Creative Commons licence – details of these licences and which Creative Commons licence will apply to this Work are set out in our licence referred to above.

Other than as permitted in any relevant BMJ Author's Self Archiving Policies, I confirm this Work has not been accepted for publication elsewhere, is not being considered for publication elsewhere and does not duplicate material already published. I confirm all authors consent to publication of this Work and authorise the granting of this licence.

For peer review only

Title:

Childhood peer status and circulatory disease in adulthood: a prospective cohort study in Stockholm, Sweden

Authors:

Alexander Miething and Ylva B Almquist

Affiliations

Department of Public Health Sciences, Stockholm University, SE-106 91, Stockholm, Sweden.

Corresponding author:

Alexander Miething

E-mail: alexander.miething@su.se

For peer review only

For peer review only - http://bmjopen.bmj.com/site/about/guidelines.xhtml

Abstract

Objectives: Childhood conditions have been recognised as important predictors of short-term and long-term health outcomes, but few studies have considered status position in the peer group as a possible determinant of adult health. Lower peer status, which often reflects experiences of marginalisation and rejection by peers, may impose inequality experiences and leave long-lasting imprints on health. The present study therefore aimed to examine whether peer status is associated with the risk for circulatory disease across adulthood.

Design: Prospective cohort study based on the Stockholm Birth Cohort Multigenerational Study (SBC Multigen).

Setting: Stockholm metropolitan area

Participants: All individuals who were born in 1953 and resident in the greater metropolitan area of Stockholm in 1963 (n=14,608). The analytical sample consisted of 5410 males and 5990 females. Peer status was sociometrically assessed when cohort members at age 13. The survey material was linked to inpatient-care registers that contained information about circulatory diseases (n=1,668) across ages 20-63. Cox proportional hazard models were used for the analysis.

Outcome measures: Circulatory disease

Results: Peer marginalisation at age 13 showed significantly higher risks of circulatory disease in adulthood among males (HR=1.34; 95% CI 1.09–1.64) and females (HR=1.33; 95% CI 1.04–1.70) alike. A graded relationship between peer status and circulatory diseases was detected in females (p=0.023). Among males, there was a threshold effect, showing that only those in the lowest status position had significantly increased risks of circulatory disease. The associations remained significant after adjusting for various conditions in child- and adulthood.

Conclusions: This study demonstrated that circulatory diseases in adulthood may be traceable to low peer status and marginalisation in childhood. It is suggested that peer status in late childhood may precede social integration in adolescence and adulthood, acting as a long-term stressor that contributes to circulatory disease through biological, behavioural, and psychosocial pathways.

For peer review only

For peer review only - http://bmjopen.bmj.com/site/about/guidelines.xhtml

Strengths and limitations of this study

- The longitudinal data material with linkages to register data allowed us to study the long-term implications of childhood conditions for health outcomes in adulthood.
- A relatively long follow-up period from 1973 to 2016 was considered.
- Peer status in childhood as well as other covariates were assessed at a single time point.
- Despite the use of a rich set of covariates, information on certain childhood adversities (e.g., child maltreatment and peer victimisation) was unavailable.
- There was little information available about health and health behaviours in childhood and across the subsequent life course that might explain the demonstrated associations.

Keywords

Peer status; Childhood; Circulatory disease; Inpatient care; Life course; Birth cohort

For peer review only

Declarations

Acknowledgement

This work was supported by the Swedish Council for Health, Working Life and Social Research [grant no. 2016-07148], (http://www.forte.se/en/).

Authors' contributions

AM and YBA conceived this study. YBA prepared and compiled the data. AM performed the statistical analyses. AM and YBA wrote the manuscript. Both authors approved the final version of the manuscript.

Competing interests

The authors declare that they have no competing interests.

Availability of data and materials

The datasets generated and/or analysed during the current study are not publicly available due to ethical regulations regarding the Stockholm Birth Cohort (SBC) study but are available from the co-author on reasonable request.

Ethics approval and consent to participate

The study was approved by the Stockholm Regional Ethics Committee (reg. no. 2016/481-31/5; 2016/481-31/5). The need for consent was waived due to the retrospective nature of this study.

For peer review only

Introduction

Innumerable studies have demonstrated the importance of childhood conditions for later life outcomes and, within health inequality research, parental socioeconomic conditions are often put forward as key determinants for adult health [1–3]. For example, adverse socioeconomic circumstances in childhood have been shown to predict poor mental health, but also metabolic problems, cardiovascular diseases, and higher mortality risks [4–6]. Children nevertheless also hold positions in status structures outside the family, such as in their peer group and school contexts. Yet, relatively few studies have addressed how experiences tied to these childhood-specific types of social structure are related to health outcomes in later life [7,8]. The present study therefore aimed to investigate the long-term implications of the child's own social position – peer status – for the risk of circulatory disease, which represents one of the leading mortality causes.

Peer orientation significantly increases in early adolescence [9]. Seeking for greater autonomy, children distance themselves from their parents and simultaneously intensify their interactions with peers [10,11]. Accordingly, the role of peer- and school-related factors become increasingly important. Intensified peer orientation has also been hypothesised to have buffering effects on socioeconomic inequalities in health. In conjuncture with the "processes of equalisation" hypothesis, the socioeconomic gradient in health tends to attenuate during the child–youth transition, while experiences related to peer interactions and youth culture become more salient [12]. A plausible explanation is that peer interactions and school environment impose a homogenising effect on children's background characteristics and supersede the repercussions of children's socioeconomic origin. Instead of socioeconomic conditions, the peer context and children's position in the peer group emerge as important determinants of health and for the adoption of health behaviours. Several studies have shown that the initiation of health-related behaviours, such as smoking, is substantially driven by peers rather than determined by socioeconomic conditions [13–15].

Peers function as socialising agents and are important for children's social, emotional, and behavioural development. Interactions with peers facilitate experiences that parents cannot supply [16], and contribute to identity formation [17] and personality maturation [18]. Furthermore, these interactions have the potential to increase children's self-worth, the ability to build relationships in later life, and adaptive adjustment [19,20]. Children are usually well aware of the advantages related to higher status in the peer group [21]. Moreover, their concerns about popularity and reputation significantly increase when they approach adolescence [22]. These affection-based dimensions play an important role in the formation of sociometric peer status, which represents an established instrument to assess the degree to which child is a liked, accepted, and integrated member of the peer group [7,23].

For peer review only - http://bmjopen.bmj.com/site/about/guidelines.xhtml

Marginalisation and peer rejection – conditions that are reflected by a low peer status position – deprive children of experiences that are important for their subsequent emotional and cognitive development, and may thus increase the risk for poor well-being and reduced mental health [24]. Östberg and Modin [25] demonstrated a graded inverse relationship between peer status and long-standing illness. Accordingly, although disadvantages arise most clearly for children in the bottom group, they are also tangible for those with higher ranks in the peer hierarchy. However, it is rather obvious that marginalised and socially isolated children suffer the most as they are exceedingly vulnerable to bullying and other forms destructive peer interactions [26]. Because (pre)adolescence coincides with an increased developmental need and strong desire for peer affiliations, peer rejection and marginalisation may not only have pervasive impacts on current well-being and health, but also affect children's ability to engage in social relationship later on [21,25,27]. As acknowledged through the theory of cumulative disadvantages [28,29], adverse conditions and negative experiences in childhood tend to accrue across the life course, and magnify detriments in later health as well [30].

Among adults, stronger social integration has been linked to reduced risks of hypertension, cardiovascular diseases, obesity, and mortality [31–33], indicating that psychosocial mechanisms are at play. It is reasonable to assume that similar processes link children's peer status to later circulatory diseases [34]. Higher status positions in the peer group enable children to derive greater emotional, cognitive, and social support from social contacts and facilitate the access to tangible and non-tangible resources provided by social contacts. In line with the buffering hypothesis of social support [35], supportive social ties impose indirect protective effects on health as they enhance individuals' coping ability and moderate their neuroendocrine or behavioural responses to acute or chronic stress [36–38]. High peer status may also directly enhance individuals' self-care, achievements, and health behaviours – factors that might mediate the link to circulatory diseases in adulthood [39]. Confounding factors may nevertheless induce spurious associations between peer status and circulatory disease in later life. Plausible confounding variables are aggressive or controversial behaviours and lower cognitive abilities that are associated with peer rejection as well as poor adult health. Likewise, family-related circumstances such as number of siblings and birth order/sibling position possibly influence children's standing in the peer group, and may also impose long-term effects on health.

Based on data from a Swedish cohort born in 1953, who have been followed for more than 60 years, the present study examines the association between sociometrically assessed peer status in childhood (age 13) and circulatory disease during adulthood (ages 20-63). Drawing on earlier research that has demonstrated socioeconomic inequalities in the risks for cardiovascular and circulatory diseases, acting through psychosocial mechanisms in the adult population, an inverse association between peer status and circulatory diseases is hypothesised. In comparison to an earlier study that used the same

For peer review only - http://bmjopen.bmj.com/site/about/guidelines.xhtml

register-linked survey material [7], the present study considers a substantially longer follow-up time and a larger set of covariates to examine the influence of third variables.

Data and methods

The data material used in the current study was the Stockholm Birth Cohort Multigenerational Study (SBC Multigen). In 2018/2019, the SBC Multigen was established through a probability matching between two longitudinal, anonymised data materials. The first was the Stockholm Metropolitan Study (SMS), defined as all individuals who were born in 1953 and resident in the greater metropolitan area of Stockholm in 1963 (n=15,117). It encompasses survey and register information collected at several time points from 1953 up until 1986, after which data were anonymised. The second was RELINK53, defined as all individuals born in 1953 who lived in Sweden in 1960, 1965, and/or 1968, as well as their ascendant, contemporaneous, and descendant family members (n=2,390,753). RELINK53 contains detailed information collected from a variety of administrative registers between 1960 and 2018. An algorithm based on a set of variables present in both data materials, rendered it possible to match 14,608 of the SMS cohort members to RELINK53 (for more information, see Almquist et al [40]). Accordingly, we are able to follow these individuals from birth up until their 60s. The Regional Ethical Review Board in Stockholm approved the creation of RELINK53 as well as the probability matching to the SMS that resulted in SBC Multigen (no. 2017/34-31/5; 2017/684-32).

Dependent variable

Information on circulatory diseases was drawn from the Swedish hospital discharge register, covering the years 1973 to 2016. This register records all inpatient care events (with overnight stays) in Swedish hospitals. In this study, circulatory diseases comprised all diagnoses included in the International Classification of Diseases (ICD 10th revision; chapter IX (I00 – I99)). The diagnoses were primarily based on judgments of medical doctors. Preceding versions of the ICD (8th and 9th revision) apply to diagnoses made from 1973 to 1986, and 1987 to 1996 respectively. These older classifications were harmonised in accordance with the 10th revision of the ICD. The using variable operationalised any events of circulatory disease occurring between 1973 and 2016. If multiple events occurred, only the first was taken into account.

Explanatory variable

Peer status was measured through the question “Whom do you best like working with at school?” [7,41]. Students were asked to nominate three classmates, in no particular order. This sociometric test was conducted among all 6-grade students in the Stockholm metropolitan area in 1966, with the

For peer review only - http://bmjopen.bmj.com/site/about/guidelines.xhtml

exception of school classes containing students with learning disabilities. Nominations from classmates who attended grade 6 but were not part of the cohort were also registered. Cohort members' mean age was 12.9 years (SD=0.29). For the purpose of the current study, we additionally excluded those who attended school classes with less than 10 children, since the peer status distribution tends to be somewhat different in small classes. Based on the number of received nominations, four categories were created: "marginalised" denoted zero nominations, "low status" one nomination, "medium status" two or three nominations, and "high status" four or more nominations.

Covariates

The covariates included family-related, school-related, and individual circumstances and conditions. The family-related factors considered the presence of siblings, parents' education and socioeconomic conditions, parents' psychiatric problems, and conditions during delivery. Number of siblings and sibling position were assessed in 1972. The latter measured whether the study participant was the only child in the family, or whether he/she was last born, middle born, or first born. Parental educational level accounted for whether there was at least one adult in the household with an upper secondary school degree. Parents' receipt of social welfare benefits accounts for socioeconomic difficulties and was available from the social registers, covering the period 1953 to 1965. Parental psychiatric problems (available from 1953 to 1972) were derived from the social registers and measured whether fathers or mothers showed symptoms of mental illness or psychiatric problems. Information on intervention during the delivery was based on delivery records. Cognitive ability can be regarded as individual-level confounder that affects a child's position in the peer group but also predicts adverse health behaviours and the risk of poor health in later life. A test of cognitive ability was conducted in 1966 and measured participants' verbal, spatial, and numerical competences. Test scores (with a possible range from 0 to 120) were grouped into quartiles of cognitive ability. Average school marks measured study participants' school performance in the 6th grade. Average teacher-reported conduct marks assessed in the 6th grade as well as crimes of violence recorded between 1962 and 1972 indicate participants' externalising problems. Similar to school marks, conduct marks and violent behaviour may act as third variable in the hypothesised association between peer status and circulatory disease. Mental disorders (ICD-10; chapter V (F00 – F09; F2 – F6; F70; F99)) and disorders due to alcohol use (ICD 10; chapter V (F10)) manifested in adulthood were drawn from register-based inpatient-care data (recorded from 1973 to 2016). Both measures were coded as binary variables and indicate whether a diagnosis was registered at least once between 1973 and 2016.

Study population

For peer review only - http://bmjopen.bmj.com/site/about/guidelines.xhtml

The study population included all cohort members in the SBC Multigen who attended school classes with at least 10 children, and had complete information on the variables of interest. Accordingly, the analytical sample resulted in 5,410 males and 5,990 females.

Statistical methods

Sex-specific cox proportional hazard models were used to estimate the risks of circulatory disease. As information on circulatory morbidity was available from 1973 to 2016, study subjects were left-truncated at 1 January 1973. Right-censoring was applied when the observation ended on 31 December 2016, or earlier, if subjects had died from any disease. Hierarchical regression was chosen as modelling strategy. Peer status was entered as single predictor in Model 1. Family-related variables were successively added to the Models 2 to 4. Model 4 additionally included individuals' cognitive ability. Model 5 also controlled for school and conduct marks, as well as crimes of violence. Finally, Model 6 further considers mental problems and disorders due to alcohol use that were diagnosed in adulthood. All analyses were performed with Stata (version 15.1).

Patient and public involvement

No patients or members of the public were involved in the study design or conduct of this study.

Results

The descriptive statistics are shown in Table 1 and illustrate a higher prevalence of circulatory diseases for males (18.5 %; n=999) as compared to females (11.2 %; n=669). The distribution of peer status is relatively similar across the sexes, although a slightly higher proportion of males (31.1 %; n=1,788) than females (28.6 %; n=1,710) were in high status positions, at the same time as more females (15.7 %; n=940) than males (12.1 %; n=652) experienced marginalisation.

< Table 1 about here >

The results from the Cox proportional hazard models are presented in Table 2 for males and in Table 3 for females. The unadjusted hazard ratios of males' peer status in Model 1 (Table 2) show that only those who are in a marginalised position have an increased risk for circulatory disease (HR=1.54; 95% CI 1.27–1.87). Controlling for sibling status and number of siblings in Model 2 does not affect the estimates. When adding parental educational level and social welfare receipt to the model, a small decrease of the hazard ratio for those in marginalised peer positions can be observed (Model 3). A likelihood ratio test confirmed that the decrease is significant compared to Model 2. The estimates are further attenuated when we adjust for parental psychiatric problems, intervention during delivery,

For peer review only - http://bmjopen.bmj.com/site/about/guidelines.xhtml

and cognitive ability in Model 4. Model 5 additionally considers conduct marks, average school marks, and crimes of violence. Although the hazard ratio decreases, a significant association between marginalisation and circulatory disease is retained. This association also persists when accounting for mental disorders and disorders due to alcohol misuse in Model 6.

< Table 2 about here >

Table 3 presents the corresponding analysis for females. Model 1 illustrates a graded association between peer status and circulatory disease. Females with medium peer status have a 28% higher risk of being hospitalised due to circulatory disease as compared to high status females. The respective increase by low peer status is 42%, and 61% for females in a marginalised position. As shown for males, a gradual attenuation of the hazard ratios of peer status can be observed when control variables are successively added to the model. In Model 4, the hazard ratio for medium status is no longer significant, whereas in Model 5, only marginalised females show a significant risk for circulatory disease (HR=1.36; 95% CI 1.07–1.74). A similar pattern with a somewhat lowered risk of circulatory disease in marginalised females is shown in Model 6 (HR=1.33; 95 % CI 1.04–1.70). Despite the non-significant estimates of medium and low status, a significant trend in the association between peer status and circulatory disease can still be detected (p=0.023). Whereas females' medium and low status positions indicate larger relative risks of circulatory diseases compared to males, marginalised peer status reveals remarkably similar estimates for males and females.

< Table 3 about here >

Discussion

The present study demonstrated that peer status in preadolescence is associated with circulatory diseases in adulthood. More specifically, being in a marginalised position in the peer group is significantly associated with increased risks of circulatory disease in both males and females. In addition, a graded association is detected for females: the lower the peer status, the higher the risk of circulatory disease. Compared to the most popular females, even those with a medium-high position in the peer group run higher risks of circulatory diseases in later life. The corresponding analyses of males revealed a threshold effect. Only socially isolated (marginalised) males displayed significantly higher hazards of circulatory disease. The demonstrated associations remained significant also after controlling for variables that could be regarded as potential confounders, including family-related factors and socioeconomic circumstances. Adjustments for school achievements (measured with school marks) and misconduct (measured with conduct marks and crimes of violence) reduced

For peer review only - http://bmjopen.bmj.com/site/about/guidelines.xhtml

For peer review only

the strength of association to some, but still a non-significant extent. Mental disorders and disorders due to alcohol misuse in adulthood are likely influenced by adversities during childhood [42,43]. In line with earlier studies, these factors were demonstrated as determinants of circulatory diseases [44,45]. Therefore, the slightly lowered magnitude in the association between peer status and circulatory disease in the female sample points to a weak third variable effect of mental health and alcohol-related problems.

Altogether, our findings suggest an increased susceptibility of marginalised peers for circulatory disease in later life, which is in line with other studies that considered long-term impacts of peer integration [4,7,31,46]. However, this remains to be confirmed with data materials that also encompass additional types of confounding and mediating factors.

Past research has argued that peer status may be considered as a childhood-specific type of social structure that in many ways resemble social status positions in adult life [7,41]. Accordingly, it is plausible that lower peer status would have health implications similar to socioeconomic deprivation and low socioeconomic position in adulthood. As also outlined earlier in this study, peer status may even equalise the effects of low parental socioeconomic position [12].

The current findings contribute to research on the long-lasting impacts of childhood circumstances on health [8,47]. Studies on health implications of adults' social relationships, however, tend to disregard the emergence of socially induced health problems and the origin of destructive social experiences at earlier stages in life (Ehrlich 2015). In line with life course theory, deficient social relationships – as well as health problems – may be the result of cumulative processes that escalate with increasing age. Accordingly, earlier research conjectured a mutual interdependence of social relationships and health at any stages in life course [25]. The results found in the current study suggest that this process can be backtracked to adolescence and childhood.

Among adults, stress-buffering effects of tangible and non-tangible support have been proposed as primary mechanisms as to why constructive social relationships are beneficial for health [35,39,48]. Low peer status, social isolation, and marginalisation may be particularly distressing in childhood. Socially isolated children suffer not only from lacking social and emotional support but also from the experience of having reduced opportunities to make friends and to control dominating behaviour by others [24]. Taken together, these adversities might disturb children's social and emotional development and lead to behaviours and disadvantages that are difficult to rectify in their later life. Other important aspects that potentially contribute to the findings in this study are peer victimisation and bullying. Such experiences often coincide with marginalisation and rejection. It is well documented that victimised children and youth have an increased propensity to develop somatic

For peer review only - http://bmjopen.bmj.com/site/about/guidelines.xhtml

problems, internalising problems, anxiety and depression disorders, and tend to take up smoking [49]. Longitudinal studies have demonstrated that such problems persist throughout adulthood, which is reflected by lower mental and physical health [50,51].

Low peer status in childhood and adolescence has been identified as source of chronic (psychosocial) stress that furthers the development of mental health problems and chronic inflammation [4,52,53]. In adulthood, the lack of social contacts is associated with disorders that develop over a long time span, including metabolic problems, hypertension, cardiovascular problems, and stroke. Earlier research on childhood conditions and life course processes [54–56], as well as the findings of the current study, suggest that the childhood perspective on the one hand and adulthood perspective on the other hand, should not be considered as independent given that socially induced morbidity and mortality in adulthood may have their origin in childhood. There is convincing evidence from neuroscience regarding how social relationships modulate neuroendocrine responses that subsequently affect the circulatory system, increasing the risk for stroke and cardiovascular diseases [57]. Similar mechanisms may apply to the findings in the current study and explain why marginalisation in the peer group poses a risk factor for the development of circulatory diseases.

Another finding worth noting are the observed differences between males and females. Similar to our results, previous studies have detected a dose-response relationship between females' peer status and metabolic syndrome in adulthood [46]. The graded associations between peer status and circulatory disease in females could suggest a greater emotional and social reactivity to chronic stress in comparison to males [58,59]. Girls' greater vulnerability to interpersonal stress may further be reflected by gender differences in social interactions and acquisition of social status in the peer group. Whereas girls tend to be relationship-oriented, boys are more involved in competitive and organised play [58]. Boys are also more concerned about achievements and problems directly affecting themselves [60]. These differences become manifest in girls' greater worries and self-blaming about others' relationship problems and concerns about negative evaluation by other peers. Because girls pay more attention to their peer environment and social interactions (Sneed & Cohen 2014), they may react more strongly to feedback from peers. In contrast, boys' stress pertains to school performance, physical competition, and close friendships rather than to their social networks. According to Oldehinkel (2007), likability and affection can be considered as the primary criteria of high peer status in girls, while admiration (of their achievements) marks the status position of boys. However, popular (high-status) peers are not necessarily well-liked, because peer status may also be achieved by dominance and aggression [61]. Another finding from the sex-specific analysis is the distinct association of crimes of violence with risks of circulatory diseases in males and females. The descriptive analysis clearly indicated that females' crimes of violence occur less frequent in

For peer review only - http://bmjopen.bmj.com/site/about/guidelines.xhtml

comparison to males, which is in line with the notion that females tend to use more indirect forms of aggression [62]. The relatively high risk for circulatory disease of females involved in violent crimes may not least reflect that those females are likely to experience serious mental health problems [63] that in turn pose a risk factor for developing circulatory diseases in later life. In fact, the somewhat lower hazard ratios of females' crimes of violence after controlling for mental health problems indicate that both conditions are linked to each other.

Strengths and limitations

A particular strength of this study is the use of longitudinal data material with linkages to register data. This allowed us to study the long-term implications of childhood conditions for health outcomes in adulthood. Participation rates were high in comparison to many other studies, which reduced the effects of selection and biases from attrition and non-response. The studied single cohort covered a fairly homogeneous population. Therefore, certain spurious effects, such as influences on the presented associations due to immigration, can be ruled out. Nevertheless, this study is also subject to limitations. Peer status in childhood as well as other covariates were assessed at a single time point. Further, there was little information available about health and health behaviours in childhood and across the subsequent life course (i.e., in adolescence and adulthood) that might affect the demonstrated associations. For example, possible third variable effects of child maltreatment, peer victimisation, smoking, and substance abuse could not be addressed in the present study. Moreover, it was not possible to determine when health problems (e.g., the predecessors of circulatory diseases) emerged. Likewise, potentially relevant information about circumstances in adulthood (e.g. achievements and ambitions) was not available in the data. It was therefore not possible to fully identify the causal pathways, for instance, whether psychological, behavioural, or biological processes contributed to demonstrated associations between peer status and circulatory disease. Despite the use of controls for parental socioeconomic status and other confounders such as family circumstances, it is nevertheless possible that selection and unmeasured confounding affected the identified associations. Given these limitations, we cannot draw any causal inference from this study.

Conclusions

This study confirmed that childhood circumstances impose health effects in later life. Peer relations do not only play an important role for children's emotional and social development but may have considerable long-term implications on their health. Congruent with earlier studies on the role of socioeconomic conditions for morbidity and mortality, the findings of this study stress the importance of children's social relationships for circulatory diseases. Our investigation of this rather specific

For peer review only - http://bmjopen.bmj.com/site/about/guidelines.xhtml

outcome might help to understand the processes that contribute to socially induced morbidity and mortality.

For peer review only

For peer review only - http://bmjopen.bmj.com/site/about/guidelines.xhtml

References

1 Cohen S, Janicki-Deverts D, Chen E, *et al.* Childhood socioeconomic status and adult health: Childhood socioeconomic status and adult health. *Ann N Y Acad Sci* 2010;**1186**:37–55. doi:10.1111/j.1749-6632.2009.05334.x

2 Galobardes B. Childhood Socioeconomic Circumstances and Cause-specific Mortality in Adulthood: Systematic Review and Interpretation. *Epidemiol Rev* 2004;**26**:7–21. doi:10.1093/epirev/mxh008

3 Poulton R, Caspi A, Milne BJ, *et al.* Association between children's experience of socioeconomic disadvantage and adult health: a life-course study. *The Lancet* 2002;**360**:1640–5. doi:10.1016/S0140-6736(02)11602-3

4 Danese A, Moffitt TE, Harrington H, *et al.* Adverse Childhood Experiences and Adult Risk Factors for Age-Related Disease: Depression, Inflammation, and Clustering of Metabolic Risk Markers. *Arch Pediatr Adolesc Med* 2009;**163**. doi:10.1001/archpediatrics.2009.214

5 Galobardes B, Smith GD, Lynch JW. Systematic Review of the Influence of Childhood Socioeconomic Circumstances on Risk for Cardiovascular Disease in Adulthood. *Ann Epidemiol* 2006;**16**:91–104. doi:10.1016/j.annepidem.2005.06.053

6 Smith GD, Hart C, Blane D, *et al.* Adverse socioeconomic conditions in childhood and cause specific adult mortality: prospective observational study. *BMJ* 1998;**316**:1631–5. doi:10.1136/bmj.316.7145.1631

7 Almquist Y. Peer status in school and adult disease risk: a 30-year follow-up study of disease-specific morbidity in a Stockholm cohort. *J Epidemiol Community Health* 2009;**63**:1028–34. doi:10.1136/jech.2009.088377

8 Caspi A, Harrington H, Moffitt TE, *et al.* Socially Isolated Children 20 Years Later: Risk of Cardiovascular Disease. *Arch Pediatr Adolesc Med* 2006;**160**:805. doi:10.1001/archpedi.160.8.805

9 Fuligni AJ, Eccles JS, Barber BL, *et al.* Early adolescent peer orientation and adjustment during high school. *Dev Psychol* 2001;**37**:28–36. doi:10.1037//0012-1649.37.1.28

10 Fuligni AJ, Eccles JS. Perceived parent-child relationships and early adolescents' orientation toward peers. *Dev Psychol* 1993;**29**:622–32. doi:10.1037/0012-1649.29.4.622

11 Starfield B, Riley A, Witt W, *et al.* Social class gradients in health during adolescence. *J Epidemiol Community Health* 2002;**56**:354–61. doi:10.1136/jech.56.5.354

12 West P, Sweeting H. Evidence on equalisation in health in youth from the West of Scotland. *Soc Sci Med* 2004;**59**:13–27. doi:10.1016/j.socscimed.2003.12.004

13 Hanson MD, Chen E. Socioeconomic Status and Health Behaviors in Adolescence: A Review of the Literature. *J Behav Med* 2007;**30**:263–85. doi:10.1007/s10865-007-9098-3

14 Kobus K. Peers and adolescent smoking. *Addiction* 2003;**98**:37–55. doi:10.1046/j.1360-0443.98.s1.4.x

For peer review only - http://bmjopen.bmj.com/site/about/guidelines.xhtml

15 Richter M, Lampert T. The role of socioeconomic status, peer and school context for adolescent smoking. *Arch Public Health* 2008;**66**:69–87.

16 Bagwell CL, Newcomb AF, Bukowski WM. Preadolescent Friendship and Peer Rejection as Predictors of Adult Adjustment. *Child Dev* 1998;**69**:140–53. doi:10.1111/j.1467-8624.1998.tb06139.x

17 Brown BB, Eicher SA, Petrie S. The importance of peer group (“crowd”) affiliation in adolescence. *J Adolesc* 1986;**9**:73–96. doi:10.1016/S0140-1971(86)80029-X

18 Reitz AK, Zimmermann J, Hutteman R, *et al.* How Peers Make a Difference: The Role of Peer Groups and Peer Relationships in Personality Development: Peers and personality development. *Eur J Personal* 2014;**28**:279–88. doi:10.1002/per.1965

19 LaFontana KM, Cillessen AHN. Children’s perceptions of popular and unpopular peers: A multimethod assessment. *Dev Psychol* 2002;**38**:635–47. doi:10.1037//0012-1649.38.5.635

20 Parker JG, Asher SR. Peer relations and later personal adjustment: Are low-accepted children at risk? *Psychol Bull* 1987;**102**:357–89. doi:10.1037/0033-2909.102.3.357

21 Lease AM, Musgrove KT, Axelrod JL. Dimensions of Social Status in Preadolescent Peer Groups: Likability, Perceived Popularity, and Social Dominance. *Soc Dev* 2002;**11**:508–33. doi:10.1111/1467-9507.00213

22 Fournier MA. Adolescent Hierarchy Formation and the Social Competition Theory of Depression. *J Soc Clin Psychol* 2009;**28**:1144–72. doi:10.1521/jscp.2009.28.9.1144

23 Parkhurst JT, Hopmeyer A. Sociometric Popularity and Peer-Perceived Popularity: Two Distinct Dimensions of Peer Status. *J Early Adolesc* 1998;**18**:125–44. doi:10.1177/0272431698018002001

24 Oldehinkel AJ, Rosmalen JGM, Veenstra R, *et al.* Being Admired or Being Liked: Classroom Social Status and Depressive Problems in Early Adolescent Girls and Boys. *J Abnorm Child Psychol* 2007;**35**:417–27. doi:10.1007/s10802-007-9100-0

25 Östberg V, Modin B. Status relations in school and their relevance for health in a life course perspective: Findings from the Aberdeen children of the 1950’s cohort study. *Soc Sci Med* 2008;**66**:835–48. doi:10.1016/j.socscimed.2007.10.018

26 Wolke D, Copeland WE, Angold A, *et al.* Impact of Bullying in Childhood on Adult Health, Wealth, Crime, and Social Outcomes. *Psychol Sci* 2013;**24**:1958–70. doi:10.1177/0956797613481608

27 Plenty S, Jonsson JO. Social Exclusion among Peers: The Role of Immigrant Status and Classroom Immigrant Density. *J Youth Adolesc* 2017;**46**:1275–88. doi:10.1007/s10964-016-0564-5

28 Dannefer D. Cumulative Advantage/Disadvantage and the Life Course: Cross-Fertilizing Age and Social Science Theory. *J Gerontol B Psychol Sci Soc Sci* 2003;**58**:S327–37. doi:10.1093/geronb/58.6.S327

29 DiPrete TA, Eirich GM. Cumulative Advantage as a Mechanism for Inequality: A Review of Theoretical and Empirical Developments. *Annu Rev Sociol* 2006;**32**:271–97. doi:10.1146/annurev.soc.32.061604.123127

For peer review only

For peer review only - http://bmjopen.bmj.com/site/about/guidelines.xhtml

30 Shuey KM, Willson AE. Economic hardship in childhood and adult health trajectories: An alternative approach to investigating life-course processes. *Adv Life Course Res* 2014;**22**:49–61. doi:10.1016/j.alcr.2014.05.001

31 Cundiff JM, Matthews KA. Friends With Health Benefits: The Long-Term Benefits of Early Peer Social Integration for Blood Pressure and Obesity in Midlife. *Psychol Sci* 2018;**29**:814–23. doi:10.1177/0956797617746510

32 House JS, Landis KR, Umberson D. Social Relationships and Health. *Science* 1988;**241**:540.

33 Valtorta NK, Kanaan M, Gilbody S, *et al.* Loneliness and social isolation as risk factors for coronary heart disease and stroke: systematic review and meta-analysis of longitudinal observational studies. *Heart* 2016;**102**:1009–16. doi:10.1136/heartjnl-2015-308790

34 Kroenke C. Socioeconomic status and health: Youth development and neomaterialist and psychosocial mechanisms. *Soc Sci Med* 2008;**66**:31–42. doi:10.1016/j.socscimed.2007.07.018

35 Cohen S, Wills TA. Stress, social support, and the buffering hypothesis. *Psychol Bull* 1985;**98**:310–57. doi:10.1037/0033-2909.98.2.310

36 Holt-Lunstad J, Smith TB, Layton JB. Social Relationships and Mortality Risk: A Meta-analytic Review. *PLoS Med* 2010;**7**:e1000316. doi:10.1371/journal.pmed.1000316

37 Seeman TE. Social ties and health: The benefits of social integration. *Ann Epidemiol* 1996;**6**:442–51. doi:10.1016/S1047-2797(96)00095-6

38 Umberson D, Karas Montez J. Social Relationships and Health: A Flashpoint for Health Policy. *J Health Soc Behav* 2010;**51**:S54–66. doi:10.1177/0022146510383501

39 Uchino BN. Social Support and Health: A Review of Physiological Processes Potentially Underlying Links to Disease Outcomes. *J Behav Med* 2006;**29**:377–87. doi:10.1007/s10865-006-9056-5

40 Almquist YB, Grotta A, Vågerö D, *et al.* Cohort profile update: The Stockholm birth cohort study. *Int J Epidemiol* Published Online First: 20 September 2019. doi:10.1093/ije/dyz185

41 Almquist Y. Social isolation in the classroom and adult health: A longitudinal study of a 1953 cohort. *Adv Life Course Res* 2011;**16**:1–12. doi:10.1016/j.alcr.2010.11.001

42 Hughes K, Bellis MA, Hardcastle KA, *et al.* The effect of multiple adverse childhood experiences on health: a systematic review and meta-analysis. *Lancet Public Health* 2017;**2**:e356–66. doi:10.1016/S2468-2667(17)30118-4

43 Shin SH, McDonald SE, Conley D. Patterns of adverse childhood experiences and substance use among young adults: A latent class analysis. *Addict Behav* 2018;**78**:187–92. doi:10.1016/j.addbeh.2017.11.020

44 Glaus J, Vandeleur C, Gholam-Rezaee M, *et al.* Atypical depression and alcohol misuse are related to the cardiovascular risk in the general population. *Acta Psychiatr Scand* 2012;:n/a-n/a. doi:10.1111/acps.12057

45 Goodwin RD, Davidson KW, Keyes K. Mental disorders and cardiovascular disease among adults in the United States. *J Psychiatr Res* 2009;**43**:239–46. doi:10.1016/j.jpsychires.2008.05.006

For peer review only - http://bmjopen.bmj.com/site/about/guidelines.xhtml

46 Gustafsson PE, Janlert U, Theorell T, *et al.* Do Peer Relations in Adolescence Influence Health in Adulthood? Peer Problems in the School Setting and the Metabolic Syndrome in Middle-Age. *PLoS ONE* 2012;**7**:e39385. doi:10.1371/journal.pone.0039385

47 Shonkoff JP, Garner AS, THE COMMITTEE ON PSYCHOSOCIAL ASPECTS OF CHILD AND FAMILY HEALTH, COMMITTEE ON EARLY CHILDHOOD, ADOPTION, AND DEPENDENT CARE, AND SECTION ON DEVELOPMENTAL AND BEHAVIORAL PEDIATRICS, *et al.* The Lifelong Effects of Early Childhood Adversity and Toxic Stress. *PEDIATRICS* 2012;**129**:e232–46. doi:10.1542/peds.2011-2663

48 Rueger SY, Malecki CK, Pyun Y, *et al.* A meta-analytic review of the association between perceived social support and depression in childhood and adolescence. *Psychol Bull* 2016;**142**:1017–67. doi:10.1037/bul0000058

49 Wolke D, Lereya ST. Long-term effects of bullying. *Arch Dis Child* 2015;**100**:879–85. doi:10.1136/archdischild-2014-306667

50 Copeland WE, Wolke D, Lereya ST, *et al.* Childhood bullying involvement predicts low-grade systemic inflammation into adulthood. *Proc Natl Acad Sci* 2014;**111**:7570–5. doi:10.1073/pnas.1323641111

51 Lereya ST, Copeland WE, Costello EJ, *et al.* Adult mental health consequences of peer bullying and maltreatment in childhood: two cohorts in two countries. *Lancet Psychiatry* 2015;**2**:524–31. doi:10.1016/S2215-0366(15)00165-0

52 Nurius PS, Green S, Logan-Greene P, *et al.* Life course pathways of adverse childhood experiences toward adult psychological well-being: A stress process analysis. *Child Abuse Negl* 2015;**45**:143–53. doi:10.1016/j.chiabu.2015.03.008

53 Yang YC, McClintock MK, Kozloski M, *et al.* Social Isolation and Adult Mortality: The Role of Chronic Inflammation and Sex Differences. *J Health Soc Behav* 2013;**54**:183–203. doi:10.1177/0022146513485244

54 Hayward MD, Gorman BK. The Long Arm of Childhood: The Influence of Early-Life Social Conditions on Men's Mortality. *Demography* 2004;**41**:87–107. doi:10.1353/dem.2004.0005

55 Miller GE, Chen E, Parker KJ. Psychological stress in childhood and susceptibility to the chronic diseases of aging: Moving toward a model of behavioral and biological mechanisms. *Psychol Bull* 2011;**137**:959–97. doi:10.1037/a0024768

56 Power C, Hertzman C. Social and biological pathways linking early life and adult disease. *Br Med Bull* 1997;**53**:210–21. doi:10.1093/oxfordjournals.bmb.a011601

57 Booth J, Connelly L, Lawrence M, *et al.* Evidence of perceived psychosocial stress as a risk factor for stroke in adults: a meta-analysis. *BMC Neurol* 2015;**15**. doi:10.1186/s12883-015-0456-4

58 Rose AJ, Rudolph KD. A Review of Sex Differences in Peer Relationship Processes: Potential Trade-offs for the Emotional and Behavioral Development of Girls and Boys. *Psychol Bull* 2006;**132**:98–131. doi:10.1037/0033-2909.132.1.98

59 Rudolph KD. Gender differences in emotional responses to interpersonal stress during adolescence. *J Adolesc Health* 2002;**30**:3–13. doi:10.1016/S1054-139X(01)00383-4

For peer review only - http://bmjopen.bmj.com/site/about/guidelines.xhtml

60 Shih JH, Eberhart NK, Hammen CL, *et al.* Differential Exposure and Reactivity to Interpersonal Stress Predict Sex Differences in Adolescent Depression. *J Clin Child Adolesc Psychol* 2006;**35**:103–15. doi:10.1207/s15374424jccp3501_9

61 Vaillancourt T, Hymel S. Aggression and social status: the moderating roles of sex and peer-valued characteristics. *Aggress Behav* 2006;**32**:396–408. doi:10.1002/ab.20138

62 Salmivalli C, Kaukiainen A. “Female aggression” revisited: Variable- and person-centered approaches to studying gender differences in different types of aggression. *Aggress Behav* 2004;**30**:158–63. doi:10.1002/ab.20012

63 Cauffman E, Lexcen FJ, Goldweber A, *et al.* Gender Differences in Mental Health Symptoms Among Delinquent and Community Youth. *Youth Violence Juv Justice* 2007;**5**:287–307. doi:10.1177/1541204007301292

For peer review only

For peer review only - http://bmjopen.bmj.com/site/about/guidelines.xhtml

1 **Table 1: Descriptive statistics for the study variables (n=11,400).**

	Males (n=5,410)		Females (n=5,990)	
	n	%	n	%
Circulatory disease (1973-2016)				
No	4,411	81.5	5,321	88.8
Yes	999	18.5	669	11.2
Peer status position (1966)				
High status	1,788	33.1	1,710	28.6
Medium status	1,937	35.8	2,185	36.5
Low status	1,033	19.1	1,155	19.3
Marginalised	652	12.1	940	15.7
Sibling position (1972)				
Only child	705	13.0	705	11.8
Last born	1,714	31.7	1,929	32.2
Middle born	1,302	24.1	1,509	25.2
First born	1,689	31.2	1,847	30.8
Number of siblings (1972)				
0	705	13.0	705	11.8
1	2,090	38.6	2,245	37.5
2	1,508	27.9	1,675	28.0
3	691	12.8	815	13.6
4	254	4.7	334	5.6
More than 4	162	3.0	216	3.6
Parental educational level (1960)				
At least one parent with upper secondary degree	1,402	25.9	1,533	25.6
No parent with upper secondary degree	3,731	69.0	4,131	69.0
Missing	277	5.1	326	5.4
Parental social welfare receipt (1953-1965)				
No	4,544	84.0	5,067	84.6
Yes	866	16.0	923	15.4
Parental psychiatric problems (1953-1972)				
No	5,105	94.4	5,644	94.2
Yes	305	5.6	346	5.8
Intervention during delivery (1953)				
No	4,090	75.6	4,580	76.5
Yes	329	6.1	293	4.9
Missing	991	18.3	1,117	18.7
Cognitive ability (1966)				
1st quartile (lowest)	1,052	19.5	1,601	26.7
2nd quartile	1,268	23.4	1,538	25.7
3rd quartile	1,491	27.6	1,509	25.2
4th quartile (highest)	1,599	29.6	1,342	22.4
Conduct marks (1966)				
Not so good	5	0.1	1	0.0
Good	83	1.5	19	0.3
Very good	5,322	98.4	5,970	99.7
Average school marks (1966)				
Range 1 to 5	Mean: 3.2	Std.dev.: 0.9	Mean: 3.3	Std.dev.: 0.9
Crimes of violence (1962-1972)				
No	5,184	95.8	5,947	99.3
Yes	226	4.2	43	0.7
Mental disorders (1973-2016)				
No	5,051	93.4	5,535	92.4
Yes	359	6.6	455	7.6
Disorders due to alcohol use (1973-2016)				
No	5,070	93.7	5,804	96.9
Yes	340	6.3	186	3.1

2

For peer review only - http://bmjopen.bmj.com/site/about/guidelines.xhtml

Table 2: The association between peer status position (1966) and circulatory disease (1973-2016) among males. Results from Cox proportional hazard regression models (n=5,410).

	Circulatory disease (1973-2016)											
	**Model 1**		**Model 2**		**Model 3**		**Model 4**		**Model 5**		**Model 6**	
	**HR**	**95% CI**	**HR**	**95% CI**	**HR**	**95% CI**	**HR**	**95% CI**	**HR**	**95% CI**	**HR**	**95% CI**
Peer status position (1966)												
High status (ref.)	1		1		1		1		1		1	
Medium status	1.13	(0.97 - 1.32)	1.12	(0.96 - 1.31)	1.11	(0.95 - 1.29)	1.08	(0.92 - 1.26)	1.06	(0.90 - 1.24)	1.05	(0.89 - 1.23)
Low status	1.14	(0.95 - 1.37)	1.13	(0.94 - 1.36)	1.11	(0.93 - 1.33)	1.07	(0.89 - 1.28)	1.03	(0.85 - 1.24)	1.01	(0.84 - 1.22)
Marginalised	1.54***	(1.27 - 1.87)	1.53***	(1.26 - 1.86)	1.49***	(1.23 - 1.81)	1.41***	(1.16 - 1.72)	1.34**	(1.10 - 1.64)	1.34**	(1.09 - 1.64)
Sibling position (1972)												
Only child (ref.)			1		1		1		1		1	
Last born			0.87	(0.70 - 1.09)	0.89	(0.71 - 1.11)	0.92	(0.73 - 1.15)	0.92	(0.74 - 1.16)	0.93	(0.74 - 1.17)
Middle born			0.82	(0.61 - 1.09)	0.83	(0.62 - 1.11)	0.86	(0.64 - 1.15)	0.86	(0.64 - 1.15)	0.88	(0.66 - 1.18)
First born			0.85	(0.67 - 1.06)	0.86	(0.69 - 1.09)	0.87	(0.70 - 1.10)	0.88	(0.70 - 1.11)	0.89	(0.71 - 1.12)
Number of siblings (1972)			1.06	(0.99 - 1.13)	1.04	(0.97 - 1.11)	1.03	(0.96 - 1.10)	1.03	(0.96 - 1.10)	1.02	(0.95 - 1.09)
Parental educational level (1960)												
At least one parent with upper secondary degree (ref.)					1		1		1		1	
No parent with upper secondary degree					1.03	(0.88 - 1.19)	0.98	(0.84 - 1.14)	0.97	(0.83 - 1.13)	0.97	(0.83 - 1.14)
Missing					1.00	(0.73 - 1.35)	0.90	(0.65 - 1.25)	0.90	(0.65 - 1.24)	0.91	(0.66 - 1.25)
Parental social welfare receipt (1953-1965)												
No (ref.)					1		1		1		1	
Yes					1.32**	(1.12 - 1.56)	1.29**	(1.08 - 1.54)	1.24*	(1.04 - 1.48)	1.21*	(1.01 - 1.45)
Parental psychiatric problems (1953-1972)												
No (ref.)							1		1		1	
Yes							1.06	(0.81 - 1.38)	1.05	(0.81 - 1.37)	1.00	(0.77 - 1.31)
Intervention during delivery												
No (ref.)							1		1		1	
Yes							1.35*	(1.06 - 1.72)	1.35*	(1.06 - 1.72)	1.35*	(1.06 - 1.72)
Missing							1.14	(0.96 - 1.35)	1.13	(0.95 - 1.34)	1.13	(0.95 - 1.34)
Cognitive ability (1966)												
1st quartile (lowest) (ref.)							1		1		1	
2nd quartile							0.90	(0.75 - 1.07)	0.91	(0.76 - 1.09)	0.94	(0.78 - 1.13)
3rd quartile							0.81*	(0.68 - 0.98)	0.83*	(0.69 - 1.00)	0.86	(0.72 - 1.04)
4th quartile (highest)							0.77**	(0.63 - 0.93)	0.79*	(0.65 - 0.95)	0.82*	(0.68 - 1.00)
Conduct marks (1966)									0.65*	(0.46 - 0.92)	0.68*	(0.48 - 0.96)
Average school marks (1966)									0.97	(0.90 - 1.03)	0.96	(0.90 - 1.03)
Crimes of violence (1962-1972)												
No (ref.)									1		1	
Yes									1.59**	(1.23 - 2.07)	1.44**	(1.10 - 1.87)
Mental disorders (1973-2016)												
No (ref.)											1	
Yes											1.43**	(1.14 - 1.79)
Disorders due to alcohol use (1973-2016)												
No (ref.)											1	
Yes											1.74***	(1.39 - 2.17)

HR=Hazard Ratio; CI=Confidence Interval; * = p<0.05; ** = p<0.01; *** = p<0.001

For peer review only

Table 3: The association between peer status position (1966) and circulatory disease (1973-2016) among females. Results from Cox proportional hazard regression models (n=5,990).

	Circulatory disease (1973-2016)											
	Model 1		Model 2		Model 3		Model 4		Model 5		Model 6	
	HR	95% CI	HR	95% CI	HR	95% CI	HR	95% CI	HR	95% CI	HR	95% CI
Peer status position (1966)												
High status (ref.)	1		1		1		1		1		1	
Medium status	1.28*	(1.05 - 1.57)	1.27*	(1.03 - 1.55)	1.24*	(1.01 - 1.51)	1.20	(0.98 - 1.48)	1.18	(0.96 - 1.45)	1.18	(0.96 - 1.45)
Low status	1.42**	(1.13 - 1.79)	1.40**	(1.11 - 1.76)	1.36**	(1.08 - 1.71)	1.30*	(1.03 - 1.64)	1.26	(0.99 - 1.59)	1.24	(0.98 - 1.57)
Marginalised	1.61***	(1.27 - 2.03)	1.54***	(1.22 - 1.95)	1.49***	(1.18 - 1.89)	1.42**	(1.11 - 1.81)	1.36*	(1.07 - 1.74)	1.33*	(1.04 - 1.70)
Sibling position (1972)												
Only child (ref.)			1		1		1		1		1	
Last born			0.76*	(0.58 - 1.00)	0.81	(0.62 - 1.07)	0.82	(0.62 - 1.07)	0.82	(0.63 - 1.08)	0.83	(0.63 - 1.10)
Middle born			0.66*	(0.47 - 0.92)	0.69*	(0.50 - 0.97)	0.68*	(0.49 - 0.96)	0.69*	(0.49 - 0.97)	0.70*	(0.50 - 0.98)
First born			0.64**	(0.48 - 0.84)	0.68**	(0.51 - 0.90)	0.69**	(0.52 - 0.91)	0.69*	(0.53 - 0.92)	0.70*	(0.53 - 0.93)
Number of siblings (1972)			1.12***	(1.05 - 1.19)	1.10**	(1.03 - 1.17)	1.10**	(1.03 - 1.17)	1.10**	(1.03 - 1.18)	1.10**	(1.03 - 1.18)
Parental educational level (1960)												
At least one parent with upper secondary degree (ref.)					1		1		1		1	
No parent with upper secondary degree					1.53***	(1.25 - 1.88)	1.49***	(1.21 - 1.84)	1.48***	(1.20 - 1.82)	1.48**	(1.20 - 1.82)
Missing					1.57*	(1.10 - 2.25)	1.55*	(1.05 - 2.27)	1.54*	(1.05 - 2.26)	1.52*	(1.03 - 2.22)
Parental social welfare receipt (1953-1965)												
No (ref.)					1		1		1		1	
Yes					1.19	(0.97 - 1.45)	0.99	(0.79 - 1.24)	0.99	(0.79 - 1.23)	0.97	(0.77 - 1.21)
Parental psychiatric problems (1953-1972)												
No (ref.)							1		1		1	
Yes							1.74***	(1.31 - 2.30)	1.74***	(1.32 - 2.31)	1.68**	(1.27 - 2.23)
Intervention during delivery												
No (ref.)							1		1		1	
Yes							1.26	(0.90 - 1.76)	1.25	(0.89 - 1.74)	1.24	(0.89 - 1.73)
Missing							0.98	(0.79 - 1.22)	0.98	(0.79 - 1.21)	0.97	(0.78 - 1.21)
Cognitive ability (1966)												
1st quartile (lowest) (ref.)							1		1		1	
2nd quartile							0.88	(0.72 - 1.08)	0.89	(0.72 - 1.09)	0.91	(0.74 - 1.12)
3rd quartile							0.95	(0.77 - 1.18)	0.97	(0.78 - 1.20)	1.00	(0.81 - 1.24)
4th quartile (highest)							0.82	(0.64 - 1.04)	0.84	(0.66 - 1.06)	0.87	(0.68 - 1.11)
Conduct marks (1966)									4.56	(0.66 - 31.66)	4.01	(0.58 - 27.70)
Average school marks (1966)									0.90*	(0.83 - 0.98)	0.90*	(0.83 - 0.98)
Crimes of violence (1962-1972)												
No (ref.)									1		1	
Yes									2.20*	(1.16 - 4.14)	1.86	(0.98 - 3.53)
Mental disorders (1973-2016)												
No (ref.)											1	
Yes											1.38*	(1.07 - 1.77)
Disorders due to alcohol use (1973-2016)												
No (ref.)											1	
Yes											1.94***	(1.40 - 2.69)

HR=Hazard Ratio; CI=Confidence Interval; * = p<0.05; ** = p<0.01; *** = p<0.001

For peer review only

STROBE 2007 (v4) Statement—Checklist of items that should be included in reports of *cohort studies*

Section/Topic	Item #	Recommendation	Reported on page #
**Title and abstract**	1	(*a*) Indicate the study's design with a commonly used term in the title or the abstract	2 (line 8)
		(*b*) Provide in the abstract an informative and balanced summary of what was done and what was found	2 (lines 23 – 26)
**Introduction**			
Background/rationale	2	Explain the scientific background and rationale for the investigation being reported	5 (lines 2 – 24)
Objectives	3	State specific objectives, including any prespecified hypotheses	6 (lines 31 – 34)
**Methods**			
Study design	4	Present key elements of study design early in the paper	2 (lines 11 – 15)
Setting	5	Describe the setting, locations, and relevant dates, including periods of recruitment, exposure, follow-up, and data collection	7 (lines 5 – 16)
Participants	6	(*a*) Give the eligibility criteria, and the sources and methods of selection of participants. Describe methods of follow-up	7 (lines 6 – 15); 9 (lines 4 – 6)
		(*b*) For matched studies, give matching criteria and number of exposed and unexposed	7 (lines 5 – 16)
Variables	7	Clearly define all outcomes, exposures, predictors, potential confounders, and effect modifiers. Give diagnostic criteria, if applicable	7 (lines 23 – 31); 8 (lines 1 – 33)
Data sources/ measurement	8*	For each variable of interest, give sources of data and details of methods of assessment (measurement). Describe comparability of assessment methods if there is more than one group	7 (line 8/9); 8
Bias	9	Describe any efforts to address potential sources of bias	8 (lines 13 – 33)
Study size	10	Explain how the study size was arrived at	7 (lines 5 – 16); 9 (lines 9 – 11)
Quantitative variables	11	Explain how quantitative variables were handled in the analyses. If applicable, describe which groupings were chosen and why	9 (lines 11 – 15)
Statistical methods	12	(*a*) Describe all statistical methods, including those used to control for confounding	9 (lines 7 – 16)
		(*b*) Describe any methods used to examine subgroups and interactions	Not applicable
		(*c*) Explain how missing data were addressed	9 (lines 4, 5)
		(*d*) If applicable, explain how loss to follow-up was addressed	Not applicable
		(*e*) Describe any sensitivity analyses	Not applicable

For peer review only

**Results**			
Participants	13*	(a) Report numbers of individuals at each stage of study—eg numbers potentially eligible, examined for eligibility, confirmed eligible, included in the study, completing follow-up, and analysed	7 (lines 7 – 11); 8 (lines 2 – 4); Table 1
		(b) Give reasons for non-participation at each stage	7 (lines 7 – 12); 8 (lines 2 – 4)
		(c) Consider use of a flow diagram	Not applicable
Descriptive data	14*	(a) Give characteristics of study participants (eg demographic, clinical, social) and information on exposures and potential confounders	7 (lines 5 – 17); 8; 9 (lines 1 – 16); Table 1
		(b) Indicate number of participants with missing data for each variable of interest	Table 1
		(c) Summarise follow-up time (eg, average and total amount)	9 (lines 9 – 11)
Outcome data	15*	Report numbers of outcome events or summary measures over time	9 (line 20)
Main results	16	(*a*) Give unadjusted estimates and, if applicable, confounder-adjusted estimates and their precision (eg, 95% confidence interval). Make clear which confounders were adjusted for and why they were included	9 (lines 11 – 15); Table 2 and 3
		(*b*) Report category boundaries when continuous variables were categorized	8 (line 27)
		(*c*) If relevant, consider translating estimates of relative risk into absolute risk for a meaningful time period	Not applicable
Other analyses	17	Report other analyses done—eg analyses of subgroups and interactions, and sensitivity analyses	Not applicable
**Discussion**			
Key results	18	Summarise key results with reference to study objectives	14 (lines 8 – 12)
**Limitations**			
Interpretation	20	Give a cautious overall interpretation of results considering objectives, limitations, multiplicity of analyses, results from similar studies, and other relevant evidence	11 (lines 2 – 18); 12; 13
Generalisability	21	Discuss the generalisability (external validity) of the study results	13 (lines 21 – 26)
**Other information**			
Funding	22	Give the source of funding and the role of the funders for the present study and, if applicable, for the original study on which the present article is based	4 (lines 3, 4)

*Give information separately for cases and controls in case-control studies and, if applicable, for exposed and unexposed groups in cohort and cross-sectional studies.

For peer review only

Note: An Explanation and Elaboration article discusses each checklist item and gives methodological background and published examples of transparent reporting. The STROBE checklist is best used in conjunction with this article (freely available on the Web sites of PLoS Medicine at http://www.plosmedicine.org/, Annals of Internal Medicine at http://www.annals.org/, and Epidemiology at http://www.epidem.com/). Information on the STROBE Initiative is available at www.strobe-statement.org.

For peer review only
